# Cyber Attacks Detection in IoT Networks Using a Distributed Inflated-Mask Encoder-Only Informer Model

**DOI:** 10.3390/s26206443

**Published:** 2026-10-12

**Authors:** Roland N. Mfondoum, Antoni Ivanov, Nikol Gotseva, Atanas Vlahov, Pavlina Koleva, Vladimir Poulkov

**Affiliations:** 1 Faculty of Telecommunications, Technical University of Sofia, Bul. Kl. Ohridski 8, 1000 Sofia, Bulgaria; mfondoum@tu-sofia.bg (R.N.M.);; 2 Intelligent Communication Infrastructure Laboratory, Sofia Tech Park, 1784 Sofia, Bulgaria

**Keywords:** anomaly detection, cyber attack detection, informer network, IoT security, CNN, statistical masking, multi-class Classification, edge computing

## Abstract

The number of Internet of Things (IoT) devices has been growing rapidly, driven by the demand for low-cost and low-power devices across a wide range of industrial and consumer applications. This expansion has broadened the attack surface exposed to cyber threats, making the detection of malicious activity in the IoT infrastructure a pressing research challenge. Most existing approaches are limited to binary anomaly detection, rely on a single data modality, or incur a computational overhead incompatible with realistic deployment on the edge. This paper introduces the Multi-modal Universal Informer Convolutional Neural Network (MUI-CNN) Classifier, a distributed architecture for multi-class attack detection across heterogeneous IoT sensor and network environments. It combines four components - a multi-modal universal data embedding layer that unifies numerical, categorical, and temporal features into a shared spatio-temporal representation, a CNN local pattern recognition layer, an encoder-only classifier for long-term temporal dependencies, and an optional statistical masking layer for greater sensitivity to low-amplitude signatures. The model is evaluated on the Telecommunication Operating Systems (OS) and Network Internet of Things (TON-IoT) dataset covering eight sensor types and ten attack classes. Results demonstrate F1 above 88% on all IoT sensors and up to 94.85% on the network sensor, confirming that each architectural component contributes a measurable and distinct improvement over partial baselines.

## 1. Introduction

The number of Internet of Things (IoT) devices requiring connection to the internet has been increasing in the current decade with the growing demand for end-user services and the variety of industrial applications requiring several different kinds of low-cost devices, with low-storage, low-power consumption, and limited computational power, to be connected [1]. This rapid proliferation has substantially expanded the attack surface accessible to malicious actors, as each connected device represents a potential entry point into the broader network infrastructure. Unlike traditional computing environments, IoT devices operate under strict resource constraints that make it impractical to deploy computationally heavy security mechanisms at the edge, creating a fundamental tension between detection capability and computational feasibility.

Detecting cyber incidents in IoT networks is a topic that has been widely covered in several research papers. The majority of existing approaches, however, address anomaly detection as a binary classification problem, flagging observations as either normal or anomalous without characterizing the nature of the threat. This limits their operational value, as distinguishing an ordinary scan from a ransomware event, or a Distributed Denial of Service (DDoS) flood from a backdoor installation attempt, requires different and time-sensitive responses that binary outputs cannot support. Additionally, IoT traffic is inherently multi-modal, combining numerical sensor readings, categorical device identifiers and protocol fields, and temporal patterns, yet most models are designed for a single data type and require significant preprocessing to handle heterogeneous inputs. This paper aims to explore the implementation of a distributed Informer model for cyber attack detection directly on individual edge IoT devices as well as centrally using network data traffic from all connected IoT sensors, addressing both the multi-class and multi-modal challenges simultaneously.

A further limitation common to Transformer-based detection models is the use of an encoder–decoder architecture, where the decoder introduces reconstruction overhead that is unnecessary when the objective is classification rather than sequence generation. Similarly, attention-based models trained on data dominated by high-amplitude spike patterns tend to focus on these easily separable events at the expense of low-rate attack classes such as scanning or injection, which produce less pronounced temporal signatures. Both limitations motivate the design choices of this paper.

This paper introduces a Multi-modal Universal Informer Convolutional Neural Network (MUI-CNN) classifier, which enhances the base Informer model introduced by Zhou et al. [2,3]. The proposed architecture yields high detection performance on IoT data and accounts for local variation in data flow as well as long-term temporal correlation, while reducing computational complexity. The proposed model is motivated by the following gaps. Existing approaches either address anomaly detection as a binary problem without differentiating attack types [4], rely on a single data modality that excludes categorical or temporal features [5,6], or introduce computational overhead through encoder–decoder reconstruction, which is unnecessary when the objective is classification [7,8]. Models that incorporate masking remain limited to univariate numerical data [9], and those that combine CNN with an Informer do not consider the advantage of removing the decoder or the effect of statistical masking on detecting the minority attack types [8,10].

This paper introduces a Multi-modal Universal Informer CNN (MUI-CNN) model that yields high detection performance on IoT data, and that considers the local variation in data flow as well as long-term temporal correlation while reducing computational complexity. This paper contributes to the field by introducing the following:

1. A **Multi-modal Universal Data Embedding layer** that blends multivariate data features—numerical, categorical, and temporal—into a unified spatio-temporal matrix, enabling a single model to operate across heterogeneous IoT sensor types without dataset-specific preprocessing.
2. A **Feature extraction and local pattern recognition layer** based on a 1D Convolutional Neural Network (Conv1D) that identifies local spatial patterns such as rapid packet bursts and short-duration anomalies within data segments, complementing the global temporal modeling of the Informer encoder.
3. An **encoder-only Informer classifier** that focuses on learning long-term patterns and temporal dependencies using Probabilistic sparse (ProbSparse) self-attention to enforce sparsity and reduce computational complexity. The decoder was eliminated to turn the encoder into a supervised learner for classification only, reducing model complexity while retaining the temporal dependency modeling capacity that distinguishes the Informer from standard Transformers.
4. An **optional statistical masking layer** embedded ahead of the encoder, applying one of three statistical masking strategies—robust dynamic Mahalanobis distance (RDMD), median absolute deviation (MAD), or Z-score—to suppress dominant spike patterns prior to encoding, thus focusing the training of the model on minor variations rather than on high-amplitude events that are already easy to detect.

Combining local pattern recognition with global-flow-level temporal dependency enables detection performance improvement across diverse sensor types. Using an Informer classifier without the decoder reduces the model’s complexity, while masking focuses Informer model training while reducing additional computational overhead. The combination of these four components is validated through the experiments, in which the deep convolutional neural network (DCNN), but with only local features, and the standalone Informer (temporal encoder only) serve as partial-component baselines against the full MUI-CNN.

## 2. Related Works

Several studies use machine learning (ML) for anomaly detection on IoT and WSN data [6,11]. The first relies on isolation forest, which outperforms K-nearest neighbor (KNN), support vector machine (SVM), and random forest (RF). The latter uses a grid-based detection approach with KNN where candidate outliers are found and maintained over several windows for deferred classification based on future patterns, abstracting temporal features.

A global probabilistic model based on the joint probability density function of all sensors constructed using the Copula method is used in [5]. Although no assumption is made on each sensor’s data distribution, it relies on a single model for outlier detection across all sensors, assuming equal environmental conditions. A variational auto-encoder (VAE) trained on normal IoT data is used for anomaly detection by [4], yielding notable accuracy but unsuitable for differentiating between the different attack types. In [7], an encoder–decoder-based anomaly scoring approach that relies on the Informer for learning long-term dependencies, is applied on intelligent transport systems data.

Several studies have explored the use of long short-term memory (LSTM) with gated recurrent units (GRUs) [12,13] or CNN for local pattern recognition [14] to detect anomalies in IoT network data. Evaluated on the TON-IoT dataset, in [15], bidirectional LSTM (BiLSTM) achieves 98.4% detection accuracy while a GRU augmented with self-attention reaches 99% with up to 84% reduction in classification time, highlighting the efficiency gains of attention-augmented recurrent models for real-time IoT intrusion detection. A bidirectional GRU (BiGRU) with attention is proposed in [16] for Industrial IoT (IIoT) intrusion detection on Edge-IIoTset and the Canadian Institute for Cybersecurity (CIC) IoT 2023 dataset, capturing temporal dependencies in both directions while handling class imbalance, and similarly in [17], where attention selectively weights the most significant timesteps in real-time IoT streams. Hybrid architectures combining BiLSTM with CNN are explored in [18,19] for IoT intrusion detection, leveraging spatial and bidirectional temporal feature extraction, while [20] combines GRU and BiLSTM on TON-IoT and CIC-IDS-2018, confirming the complementarity of gated and bidirectional memory mechanisms for multi-class attack classification. A temporal convolutional Informer is used in [10] for anomaly detection in wind turbines with RDMD and a delayed perception module for classification. Both [21,22] use a base Informer model for attack detection in power IoT, with its fixed window allowing traffic segmentation and improving computational efficiency for DDoS and intrusion detection on the CIC-IDS-2018 dataset. In [23], entropy-based pruning reduces input complexity before Informer-based label prediction, improving detection accuracy.

A graph attention transformer (GAT) is combined with an Informer model in [24] for long-term forecasting of high-dimensional data with 127 features from 51 sensors. This outperformed several baseline models, though with user-defined thresholds and in addition, the computational complexity was not considered. While [8] used a combination of CNN and an Informer model with a softmax classifier to detect cyber attacks in real-life cloud data, though without consideration of the constraints on complexity, the work [9] relied on statistical masking in the encoder based on Z-score and MAD for Informer-based outlier detection to improve detection accuracy on univariate numerical continuous data, hence reducing the computational cost. Table 1 summarizes the related works and the contributions of this paper.

## 3. Methodology

**Figure 1:**
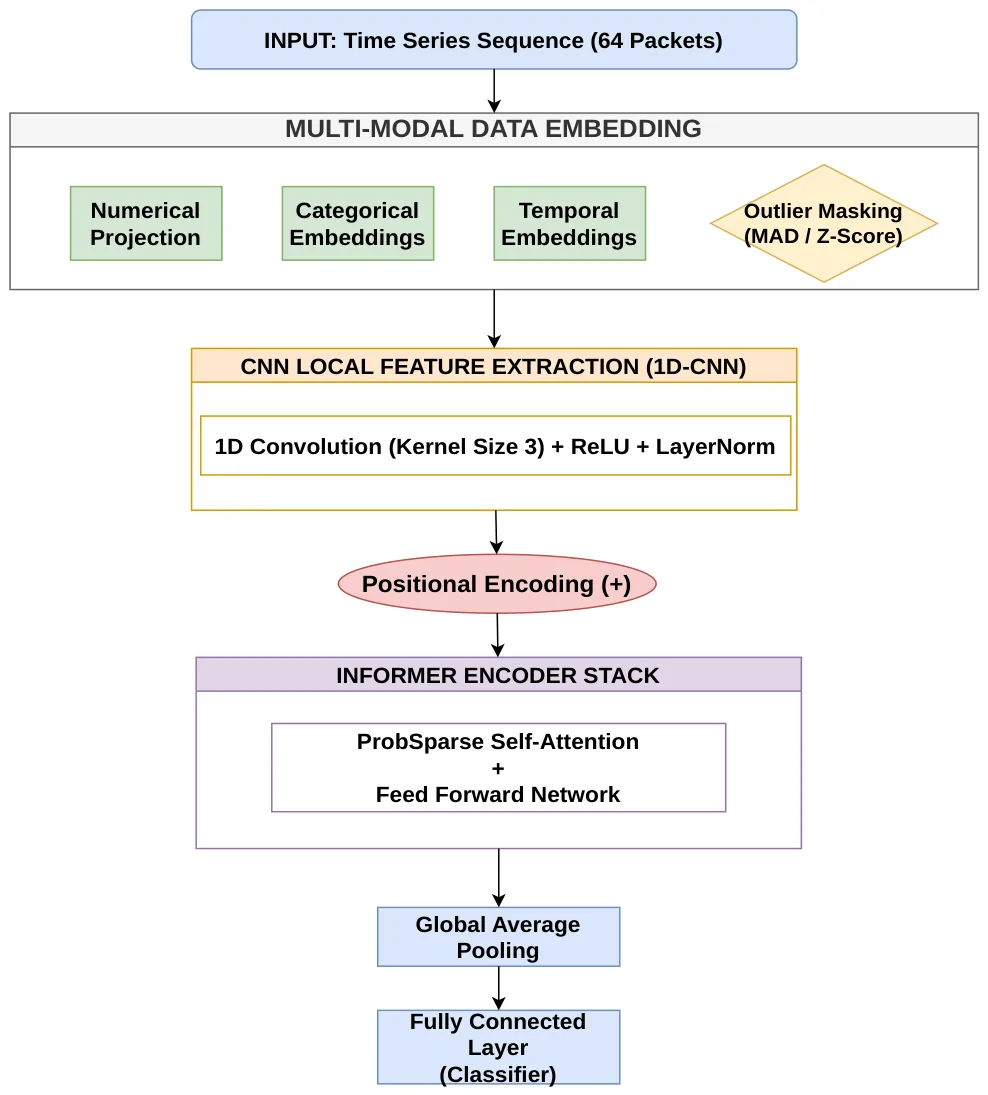
High-level building blocks of the MUI-CNN model pipeline.

**Figure 2:**
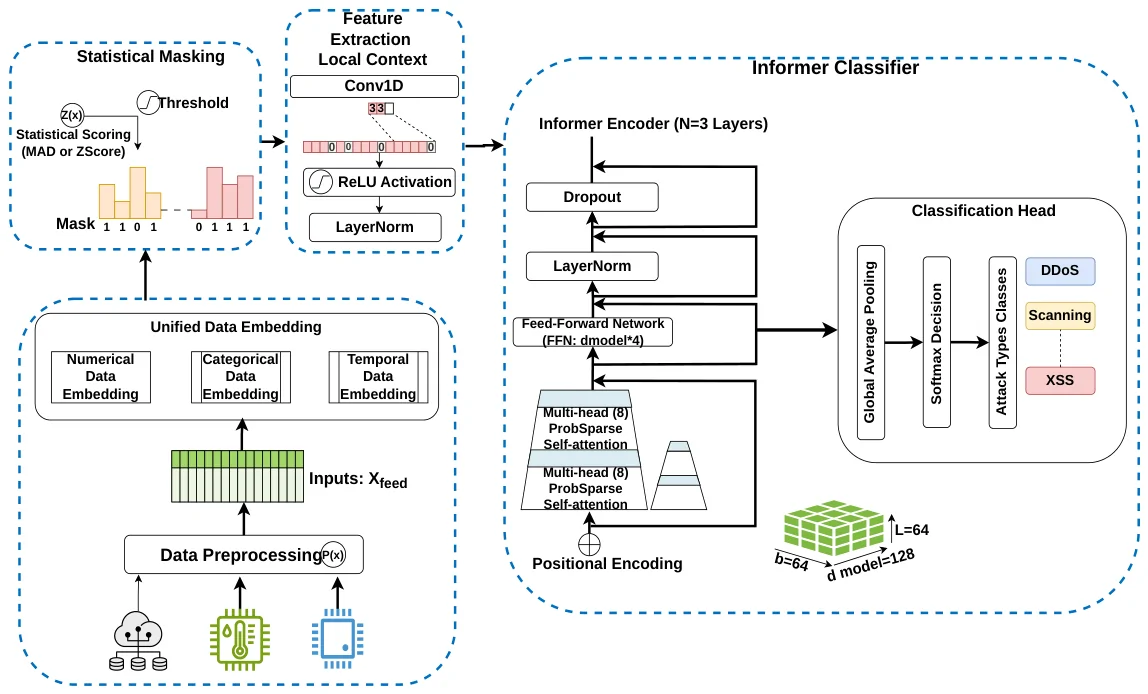
Detailed MUI-CNN architecture and data transformations pipeline.

The Multi-modal Universal Informer CNN Classifier (MUI-CNN) architecture follows a pipeline of four phases—A multi-modal embedding stage, an optional masking layer, a local feature extraction stage via 1D-CNN, and a multi-head self-attention transformer encoder classifier. This dual-extraction strategy ensures that the model is sensitive to both ephemeral attacks and long-duration malicious behaviors. Figure 1 describes the high-level building blocks of the model pipeline while Figure 2 shows the detailed major components, including data transformation when flowing through them.

### 3.1. Multi-Modal Universal Data Embedding Layer

To handle the diverse nature of IoT network traffic, the model uses a specialized input layer that processes numerical, categorical, and temporal data streams in parallel. Every packet in a sequence is represented by a high-dimensional dense vector preserving its statistical and contextual characteristics.

- **Numerical embedding.** Numerical features are projected linearly to a high-dimensional vector using a learnable weight matrix:
  (1) $$E_{\mathrm{num}}=X_{\mathrm{num}}W+b,$$
  where $X_{\mathrm{num}}$ is a *d*-dimensional numerical vector, $W\in R^{n\times d}$, and *b* the bias.
- **Categorical embedding.** Each categorical feature $X_{\mathrm{cat},i}$ with *n* classes has each class $c_{i}$ projected to a *d*-dimensional vector $M_{i}$. The final categorical embedding is as follows:
  (2) $$E_{\mathrm{cat}}=\sum_{i=1}^{n}X_{\mathrm{cat},i}.$$
- **Temporal embedding.** Temporal features are treated as periodic categorical variables and embedded identically:
  (3) $$E_{\mathrm{time}}=\sum_{i=1}^{n}T_{i}.$$
  The unified packet representation at time *t* is as follows:
  (4) $$X_{\mathrm{input}}=E_{\mathrm{num}}+E_{\mathrm{cat}}+E_{\mathrm{time}}.$$

### 3.2. Statistical Masking Layer

Statistical masking is optionally applied to $X_{\mathrm{input}}$, yielding $X_{\mathrm{masked}}=\mathrm{Masking}\left(X_{\mathrm{input}}\right)$, where the masking function sets a value to 0 if its statistical score exceeds a threshold τ, or retains the original value otherwise. Two masking functions are implemented:

- **MAD masking**:
  (5) $$x_{i,j}^{\prime }=\left\{\begin{array}{cc} 0 & \mathrm{if}\;\frac{c\;*\;|x_{i,j}-\mathrm{median}\left(x_{i}\right)|}{\mathrm{MAD}}>\tau \\ x_{i,j} & \mathrm{otherwise} \end{array}\right.$$
  with
  $$c=\Phi^{-1}\left(0.75\right)\approx 0.6745,$$
  where $\Phi^{-1}$ is the inverse standard normal cumulative distribution function, hence making the quantity $\mathrm{MAD}/\Phi^{-1}\left(0.75\right)$ a consistent estimator of σ.
- **Robust dynamic Mahalanobis distance (RDMD) masking**. The Mahalanobis distance [25] generalizes the standard deviation to multivariate settings by measuring how many standard deviations a point lies from the distribution mean, scaled by the covariance structure of the data. For a multivariate observation $x\in R^{d}$, the Mahalanobis distance from the distribution centroid μ is
  (6) $$D_{M}\left(x\right)=\sqrt{{\left(x-\mu \right)}^{⊤}\Sigma^{-1}\left(x-\mu \right)},$$
  where Σ is the covariance matrix of the distribution. Unlike Euclidean distance, $D_{M}$ accounts for correlations between dimensions and is scale invariant, making it suitable for heterogeneous feature spaces.
  In the context of streaming IoT data where statistical properties of the traffic may shift over time due to concept drift, a *robust* and *dynamic* variant is used. Robustness is achieved by replacing the sample mean and covariance with their robust counterparts—the minimum covariance determinant (MCD) estimator [26], which are resistant to the influence of outliers and do not increase the distance estimate in the presence of anomalies in the training window. The dynamic component refers to the use of a tumbling window of length *W* over the incoming data stream, such that μ and Σ are recomputed at each step on the most recent *W* observations, allowing the reference distribution to adapt to gradual changes in normal behavior:
  (7) $$D_{\mathrm{RDMD}}\left(x_{t}\right)=\sqrt{{\left(x_{t}-{\hat{\mu }}_{W}\right)}^{⊤}{\hat{\Sigma }}_{W}^{-1}\left(x_{t}-{\hat{\mu }}_{W}\right)},$$
  where ${\hat{\mu }}_{W}$ and ${\hat{\Sigma }}_{W}$ are the robust MCD estimates of the mean and covariance computed over the window ending at time *t*.
  The RDMD masking function then suppresses observations whose distance from the local robust centroid exceeds a threshold τ:
  (8) $$x_{i,j}^{\prime }=\left\{\begin{array}{cc} 0 & \mathrm{if}\;D_{\mathrm{RDMD}}\left(x_{i}\right)>\tau \\ x_{i,j} & \mathrm{otherwise}. \end{array}\right.$$
  Compared to the univariate MAD, RDMD captures inter-feature correlations and is therefore better suited to multivariate sensor streams where anomalies manifest as unusual combinations of features rather than outliers in any single dimension. Its dynamic nature also makes it more robust to gradual baseline shifts common in long-running IoT deployments, at the cost of a higher computational overhead due to the tumbling window covariance inversion.
- **Z-score masking:**
  (9) $$x_{i,j}^{\prime }=\left\{\begin{array}{cc} 0 & \mathrm{if}\;\frac{|x_{i,j}-\mathrm{mean}\left(x_{i}\right)|}{\sigma }>\tau \\ x_{i,j} & \mathrm{otherwise} \end{array}\right.$$

### 3.3. Feature Extraction and Local Context (Conv1D)

The masked data $X_{\mathrm{masked}}$ is passed through a 1D convolutional neural network (Conv1D) and a fully-connected (FC) layer for local feature extraction. In the IoT context, Conv1D detects patterns such as rapid packet bursts by applying filters acting as small tumbling windows over the data sequence. The processing pipeline is as follows:

1. Conv1D with kernel size *K* applied over sequence positions;
2. Padding to maintain fixed segment length L=64;
3. ReLU activation to learn non-linear intra-burst relationships;
4. Layer normalization to prepare data for multi-head attention.

Positional encoding $P\in R^{n\times d}$ is then added to capture the packet order. The unified representation at time *t* is

(10) $$z_{t}=\mathrm{LN}\left(\mathrm{ReLU}\left(\mathrm{Conv}1D\left(\mathrm{Mask}(\mathrm{FC}\left(x_{\mathrm{num},t}\right)+E_{\mathrm{cat},t}+T_{t}+P_{t})\right)\right)\right)$$

### 3.4. Encoder-Only Informer Classifier

The Informer classifier block, modified to retain only its encoder module, eliminating the decoder, takes $z_{t}$ as input. Attack class labels are supplied directly as output targets, making this model a supervised classifier, which is comprised by the following three components:

- **ProbSparse self-attention** uses probability distilling to identify active queries that deviate significantly from the mean, acting as second-layer spike detection after statistical masking. Computational complexity is O(LlogL), which is substantially lower than the standard attention.
- **Multi-head attention (MHA)** allows parallel processing of the *d*-dimensional latent space by splitting it across heads, each focusing on a different aspect of data (e.g., packet frequency and burst shape). This enables simultaneous assessment of multi-modal signals to identify multi-vector attack patterns.
- **A feed-forward network (FFN)** projects the *d*-dimensional vector to a higher-dimensional space ($d_{\mathrm{model}}\times 4=512$) before compressing it back with ReLU activation. A global average pooling layer then transforms each $d_{\mathrm{model}}$ vector to a scalar, followed by a Softmax function for attack-class selection.

### 3.5. Computational Complexity Evaluation

Model computational complexity is quantified using multiply-accumulate operations (MACs), where one MAC corresponds to one fused multiplication and addition. MACs are reported in millions (MMACs) per single inference window of length *L* and are computed analytically from each architecture’s layer specification, providing a hardware-agnostic measure that is independent of implementation and device. For a given input of dimension *F*, the MAC count of each layer type is derived as follows.

A **fully-connected (dense) layer** mapping $R^{F}\to R^{H}$ contributes F×H MACs. A **Conv1d layer** with $C_{\mathrm{in}}$ input channels, $C_{\mathrm{out}}$ output channels, kernel size *k*, and output length $L_{\mathrm{out}}$ contributes $L_{\mathrm{out}}\times C_{\mathrm{out}}\times C_{\mathrm{in}}\times k$ MACs. An **LSTM cell** over one timestep with input dimension $F_{t}$ and hidden dimension *H* computes four gated projections, each mapping $(F_{t}+H)\to H$, giving $4(F_{t}+H)H$ MACs per step; a **GRU cell** uses three projections at $3(F_{t}+H)H$ MACs per step; **bidirectional** variants double these counts. For a sequence of length *L* and $n_{𝓁}$ stacked layers the total gated recurrent cost is given by Equation (11):

(11) $${\mathrm{MACs}}_{\mathrm{gated}}=\sum_{𝓁=1}^{n_{𝓁}}L\cdot g\cdot \left(F_{𝓁}+H\right)\cdot H,$$

where g∈{3,4} for GRU-based and LSTM-based cells, respectively, $F_{1}=F$, $F_{𝓁>1}=H$ (or 2H for bidirectional variants), and $L_{𝓁}=L$ under the assumption of no intra-stack downsampling. **Scaled dot-product attention** (full) over sequence length *L* with model dimension *D* and $n_{h}$ heads contributes $3LD^{2}$ MACs for Q/K/V projections, $L^{2}D$ for attention score computation, and $LD^{2}$ for the output projection. The **ProbSparse attention** mechanism of the Informer replaces the $O(L^{2})$ score term with O(LlogL), reducing per-layer attention cost to

(12) $${\mathrm{MACs}}_{\mathrm{attn}}=3LD^{2}+LD\log_{2}\;L+LD^{2}$$

Each transformer encoder layer additionally contains a position-wise feed-forward network (FFN) with expansion factor *r*, contributing 2×L×D×(rD) MACs. For $n_{𝓁}$ encoder layers, the total transformer cost is $n_{𝓁}\times \left({\mathrm{MACs}}_{\mathrm{attn}}+{\mathrm{MACs}}_{\mathrm{FFN}}\right)$. The **CNN front-end** of the MUI-CNN architecture prepends a single Conv1d(*F*, *D*, k=3) block before the transformer encoder, adding L×D×F×3 MACs, masking strategies (Z-score, RDMD and MAD) operate as input preprocessing steps and introduce no additional learned parameters or MACs.

Beyond theoretical MACs, empirical resource usage is recorded for each model–sensor–fold combination during training and inference: execution time (seconds), peak CPU memory (MB), mean CPU utilization (in %), peak GPU memory (MB), and mean GPU utilization (in %). These measurements characterize practical deployment cost, while MMACs serve as the primary hardware-independent complexity metric for cross-model comparison.

## 4. Datasets Description and Processing

The model was tested on two different real-time intrusion detection system IoT datasets to ensure the ability to generalize the model to any IoT dataset sharing the same characteristics. Key requirements for the dataset selection were that they should be public, IDS and IoT related, and more importantly real-time, therefore having the timestamp feature required for Informer model training. The TON_IoT (2020–2021) and CIC IIoT (2025) datasets described below were then selected for model testing.

### 4.1. TON-IoT Dataset

The TON-IoT dataset [27] is a large-scale library produced for cyber security applications in IoT and IIoT, where TON stands for telemetry (T), operating system (O), and network (N). It comprises three components:

- **IoT telemetry**: data from seven sensors—Fridge, Garage Door, Geostationary Positioning System (GPS) Tracker, Modbus, Motion Light, Thermostat, and Weather, capturing real-time physical parameters.
- **Operating system (OS)**: Linux and Windows logs from process, memory, and network activities.
- **Network**: data captured in packet capture (PCAP) and Bro/Zeek formats, with features extracted into comma-separated values files.

Data was collected under normal operations and under various attacks: scanning, distributed denial of service (DDoS/DoS), cross-site scripting (XSS), backdoor, password brute-force, injection, ransomware, and man-in-the-middle (MITM). Both the attack type label and a binary normal/attack label are consistently recorded across all datasets. Table 2 describes the dataset structure, with the component column indicating which part of the dataset is described, the specific type of Sensor in a given dataset component, the number of records generated by a specific sensor, the number of features, and the percentage of attacks in the given data. The number of records varies from 28,000 for OS data to 22 millions for network data, which also contains the largest proportion of attacks (96.25%). Feature counts also range from six to seven features for sensor data to over 100 for network data. The combination of record counts and feature counts illustrates the variety of dimensionality in the components of this dataset.

### 4.2. CIC IIoT 2025 Dataset

The CIC IIoT Dataset 2025 [28] is a benchmark dataset produced by the Canadian Institute for Cybersecurity (CIC) at the University of New Brunswick in 2025, designed to reflect realistic Industrial Internet of Things (IIoT) environments. Benign data generated by several heterogeneous IIoT devices was collected and various attacks were performed on the related devices. Benign and attack data were then sampled at regular time intervals and integrated to produce a 10-s sample dataset featuring operations under both normal and adversarial conditions. The dataset encompasses seven attack categories—DDoS, DoS, reconnaissance, web attacks, brute force, man-in-the-middle (MITM), and malware alongside benign traffic. Each record aggregates statistical summaries of network-level features (packet sizes, inter-arrival times, TCP flags, TTL, window sizes, and IP fragmentation) and log-level features (message counts, data ranges, and data types) over the 10-s window, yielding 94 raw columns. Table 2 summarizes the structure of both datasets used in this study.

### 4.3. Data Processing

This study uses the authoritative feature-type documentation published alongside the TON-IoT dataset [27] to derive an explicit, sensor-specific feature schema for each data source. Every column in every sensor dataset is assigned a data type—number, string, Boolean, and time based on the official dataset description—and a corresponding preprocessing treatment is applied consistently across all cross-validation (CV) folds (Table 3). Features with single value or mostly missing values are considered insignificant and were discarded. All numeric features are coerced to numeric, and missing values filled with zeros. Since cross-validation was used for model training, data scaling was applied within each fold to avoid leakage. Categorical features were passed through systematic preprocessing steps, including stripping white spaces and punctuation marks, turning them to lower case to ensure consistency, and applying label encoding to variables of two classes or more. Boolean features were simply treated as numerical features. The date and time column was decomposed into six columns (type of cycles): month, weekday, day, hour, minute, and second. Sine–cosine encoding was used instead of raw integers to preserve the cyclical continuity of temporal features: hour 23 and hour 0 are adjacent in the sine–cosine space but maximally distant as integers. The raw temporal integer columns are discarded after encoding. If a temporal column contains only a single unique value across the dataset (zero variance, as occurs for month in the WIN10 dataset, which spans only April 2019), it is discarded as zero variance before encoding is attempted, and the corresponding sine or cosine columns are simply absent from the feature f.

(13) $$f\text{\_sin}\left(t\right)=\sin\;\left(\frac{2\pi t}{T}\right)$$

(14) $$f\text{\_cos}\left(t\right)=\cos\;\left(\frac{2\pi t}{T}\right)$$

Zero-variance columns (any column containing a single unique value across all rows), including columns that are entirely NotANumber (NaN), are removed universally from all sensor datasets. Such columns cannot contribute to any learned decision boundary and only increase the parameter counts of embedding tables and linear projection layers. GPS tracker sensor—the GPS tracker dataset records latitude and longitude coordinates from a simulated IoT GPS device. Analysis of the data reveals a GPS spoofing attack pattern: during normal operation the device reports a stable, tightly clustered position that varies by date. During attack periods, the coordinates systematically shift to a different cluster nearby, regardless of the date, a spoofed position being injected into the sensor stream. A key preprocessing decision concerns the derivation of engineered features. Initial analysis suggested features such as displacement between consecutive readings, velocity, rolling standard deviation of position, daily baseline deviation, and interquartile range (IQR)-based outlier flags. The final GPS feature set for cross validation consists of only three columns:

- *latitude* and *longitude*—raw coordinate values. These carry the full attack signal: attack rows consistently fall near (0,10), while normal rows cluster at the device’s actual daily location. This signal is preserved under random shuffling because the coordinate values are intrinsic to each row.
- *is_sentinel*—a binary flag set to 1 when latitude=0 and longitude=10 exactly, indicating that the device was idle or had no GPS fix. This is a row-intrinsic feature requiring no cross-row computation.

The CIC IIoT Dataset 2025 went through similar preprocessing steps to most of the TON IoT network datasets to which it is closer in nature. However, in this case, each record represents a 10-s capture window rather than an individual flow or event, so no per-flow session grouping is applied. The raw dataset contains 94 columns, of which 13 store list strings encoding per-window sets of observed IP addresses, MAC addresses, ports, and protocols, which were dropped as they could not be represented as fixed-width numeric features. Their information content is preserved through the corresponding integer count columns already present in the dataset. The device MAC address is similarly removed as a non-generalizing identifier. The column containing device names is left as a categorical sole device-level discriminator. Infinite values, which arise in ratio features during low-traffic windows, are replaced with zero. From the two timestamp columns, the start times are used as timestamp features and are parsed to a datetime object, stripped of its coordinated universal time timezone offset, and encoded into twelve sin/cos cyclic features spanning six temporal cycles (second of minute, minute of hour, hour of day, day of week, day of month, and month of year), after which the two raw timestamp columns are dropped. The original label columns are discarded and only the one containing attacks aggregated into seven types is kept as a target feature for the prediction. It is then normalized using a canonical label map that unifies spelling variants. The final representation retains 68 numeric features, one categorical feature, and 12 temporal encodings, for a total of 81 model-ready columns after one-hot expansion of the device name column.

Upon processing, training is performed using cross validation (CV) over five folds of each dataset and prediction time is measured over a small amount of test data to measure the computational performance, rather than the prediction performance largely captured in the CV. More details will be provided in the experimental setup section.

## 5. Experimental Results

### 5.1. Experimental Setup

The proposed MUI-CNN architecture was evaluated on the CIC dataset and on all sensors of the TON IoT dataset, including seven IoT sensors, two OS (Windows and Linux), and a network dataset. The Linux OS datasets were merged given that they had the same features, while the Windows 7 and 10 datasets were treated separately due to the features differences. The MUI-CNN model variants were then compared to prominent models used in the recent literature:

- **DCNN**: flat multi-layer perceptron (MLP) applied to each row independently, with no temporal encoder or sequence modeling.
- **Temporal DCNN**: same flat MLP architecture extended with cyclical sine/cosine temporal features to provide calendar context without sequence modeling.
- **BiGRU-MHA**: bidirectional GRU with an MHA layer, capturing temporal dependencies in both forward and backward directions with attention-weighted timestep importance.
- **LSTM**: unidirectional LSTM network, providing a sequential recurrent baseline for comparison against bidirectional and attention-augmented variants.
- **BiLSTM**: bidirectional LSTM network, modeling sequential attack patterns from both past and future context within each sliding window.
- **Informer**: encoder-only Informer with sparse self-attention for long-term temporal dependency modeling, without CNN preprocessing.
- **MUI-CNN**: full proposed architecture with Conv1D local pattern extraction feeding the Informer encoder, without statistical masking.
- **MUI-CNN-m**, **MUI-CNN-z**, **MUI-CNN-r**: full MUI-CNN architecture with statistical masking applied in the encoder using MAD, Z-score, and RDMD strategies, respectively.

#### 5.1.1. Training Protocol

All model variants are evaluated using stratified 5-fold cross-validation. Stratification preserves the proportional class distribution of attack types across all folds, which is essential given the pronounced class imbalance present in both datasets. In the TON-IoT dataset, normal traffic constitutes between 65% and 90% of records depending on the sensor; in the CIC IIoT 2025 dataset it accounts for approximately 45%, with the remaining share distributed across seven attack categories. Each fold uses 80% of rows for training and 20% for validation. Classification metrics are reported on the pooled predictions across all five folds, following the repeated hold-out protocol recommended for imbalanced multi-class datasets [29]. To prevent data leakage, all normalization statistics (RobustScaler parameters) are estimated exclusively from the training partition of each fold and applied to the separate validation partition.

Sequence windows are constructed using a tumbling (non-overlapping) scheme in which the slide distance *d* is set equal to the window size *s* (d=s), so that each observation contributes to exactly one window. This guarantees that no raw observation appears in both a training and a validation window, eliminating row-level leakage across fold boundaries regardless of how the stratified split assigns windows to folds. Under this protocol, global temporal ordering is not preserved across folds; temporal context is instead made available to the model through twelve sin/cos cyclic features derived from each window’s timestamp, encoding periodicity at the second, minute, hour, day-of-week, day-of-month, and month-of-year granularities.

A chronological evaluation strategy—assigning the earliest records to training and the most recent to testing—was considered but rejected for the TON-IoT dataset. Its controlled-capture methodology confines each attack type to a dedicated, non-overlapping time window [27], meaning that a strict temporal split would place entire attack classes exclusively in either the training or the test partition, rendering multi-class discrimination structurally impossible. Stratified cross-validation with tumbling windows is therefore both the methodologically appropriate and the practically necessary evaluation protocol for this dataset.

#### 5.1.2. Class Imbalance Handling

The TON-IoT dataset exhibits severe class imbalance across all sensor streams. Inverse square-root class weighting is applied to the cross-entropy loss for all models:

(15) $$\begin{array}{c} w_{c}=\frac{1}{\sqrt{n_{c}+1}},\;{\bar{w}}_{c}=\frac{w_{c}\cdot C}{\sum_{c^{\prime }}w_{c^{\prime }}}, \end{array}$$

where $n_{c}$ is the training count for class *c* with *C* being the number of classes. Inverse square-root weighting was selected over the linear variant of the same algorithm, as it provides a less aggressive penalty for the majority class (normal traffic) while still providing a sufficient gradient signal for minority attack types such as XSS, ransomware, and man-in-the-middle. Classes absent from a given training fold receive weight zero and do not contribute to the loss. For validation area under the receiver operating characteristic (AUROC) calculation, if a class is absent from the validation fold’s ground-truth labels, the probability matrix is sliced to only the present classes, renormalized row-wise, and scored to avoid the class-count mismatch error that would otherwise result from standard AUROC computation.

#### 5.1.3. Early Stopping and Learning Rate Scheduling

All models employ early stopping (ES) on validation loss with patience P=5 epochs and improvement threshold $\delta =10^{-4}$. Training terminates when the validation loss fails to decrease by more than δ for five consecutive epochs. The epoch ceiling is set to 50 for all models, while in practice, ES terminates training well before this ceiling for all sensor–model combinations tested. The checkpoint saved corresponds to the epoch achieving the highest weighted validation F1-score rather than the lowest validation loss—the two criteria operate independently so that the reported metrics reflect the best discrimination performance regardless of the exact convergence point. A patience of 5 was selected over the more commonly used value of 3 to accommodate the oscillatory convergence behavior observed under severe class imbalance. With inverse-square-root weighted loss functions, early training epochs produce unstable validation loss trajectories as the model adjusts its decision boundary for minority attack classes. A patience of 3 was found to terminate training prematurely in preliminary experiments before these boundaries had stabilized. Table 4 summarizes the most important hyperparameters used for each model training.

Detection performance is reported using overall accuracy, AUROC, and macro-averaged F1, as follows. The accuracy measures aggregate correct classification rate. The AUROC measures rank-order discrimination independent of decision threshold. Macro-averaged F1 is used in this context given that our target variable is multi-class, as opposed to the standard F1 score used for binary classification; it averages precision and recall equally across all classes regardless of frequency, and is the primary model selection criterion in this imbalanced multi-class setting.

Computational efficiency is assessed in parallel across both the training and testing (detection) phases, using four resource metrics recorded for each model—sensor pair: duration in seconds; peak central processing unit (CPU): memory in MB; average CPU: utilization in percent; and peak graphical processing unit (GPU): memory in MB. Training duration reflects the cost of fitting the model from scratch on each sensor’s dataset. Testing duration reflects the real-time inference cost—the operationally critical metric for edge-deployed anomaly detection, applied to each sensor’s test data. GPU utilization was zero across all experiments, indicating that all computation ran on the CPU, which is consistent with the resource constraints typical of IoT edge deployments. Nevertheless, the training is performed on the GPU as in realistic deployments, as it is not to be expected that IoT devices should execute this process, but rather dedicated computing nodes.

### 5.2. Overall Classification Performance

Table 5 reports cross-validation accuracy, AUROC, and macro-averaged F1 as mean ± 95% CI across 5 folds, computed using the *t*-distribution with 4 degrees of freedom and a significance level of 0.025, denoted as $t_{0.025,4}$ equaling a critical value of 2.776. If the distribution score exceeds this value, the point is classified as a possible outlier. The confidence intervals (CIs) quantify fold-to-fold variability and allow direct assessment of whether observed differences between models are statistically consistent across folds.

**IoT sensors**—Across the six simple IoT sensor datasets (**Motion Light, Fridge, Garage Door, Modbus, Thermostat, and Weather**), all MUI-CNN variants achieve consistently high performance with narrow CIs: accuracy of 95.8–99.5% ± 0.04–0.83% (range of CIs), and macro F1 of 77.0–97.2% ± 0.71–10.4%. The narrow CIs on Fridge (MUI-CNN: macro F1 of 97.21±0.71%) and Thermostat (MUI-CNN-r: macro F1 of 82.05±1.71%) confirm that MUI-CNN performance is stable across all five folds, not driven by outlier folds. In contrast, DCNN, T-DCNN, Informer, BiGRU-MHA, BiLSTM, and LSTM all displayed a macro F1 between 11.55±0.00% and 19.49%, with narrow intervals confirming that the models fall in the majority class prediction trap and collapse systematically at all folds.

**The Modbus sensor** exhibits the widest MUI-CNN CIs (MUI-CNN: macro F1 of 77.00±10.42%; MUI-CNN-z: macro F1 of 79.96±7.23%), indicating that the Modbus register code distributions vary substantially across folds, likely due to the uneven temporal concentration of certain attack types. MUI-CNN-m achieves the best and most stable performance on Modbus (macro F1 of 81.70±2.73%), with a CI approximately four times narrower than the unmasked variant, demonstrating that MAD-based attention masking actively stabilizes training across the high-variance Modbus register patterns. Among recurrent models, BiGRU-MHA reaches a macro F1 of 69.08±10.93% on Weather—the strongest recurrent result for any IoT sensor—though the wide CI reflects sensitivity to folds that capture the BiGRU’s attention mechanism aligning with the temporal attack structure. MUI-CNN-r achieves a macro F1 of 93.18±4.12% on the same sensor, confirming a statistically robust improvement of approximately 24% over the best recurrent model.

**GPS Tracker**—MUI-CNN variants achieve macro F1 of 89.2–91.2±1.3–5.5% on GPS, with MUI-CNN-r yielding the highest score, and MUI-CNN-z showing the tightest interval (90.67±1.27%), confirming stable detection of GPS coordinate cluster shifts across all folds. The Informer under performs, with a macro F1 of 19.50±3.04%. DCNN and T-DCNN achieve macro F1 scores of 69.2–69.5±0.7–0.8%, with very tight intervals, confirming that raw coordinate values carry sufficient row-level discriminative power for the non-temporal model to partially separate the coordinate clusters, though they are 22% below the best model MUI-CNN-r.

**Linux and Network sensors**—For the Linux dataset, DCNN achieves a macro F1 of 83.47±3.89%. However, the perfect AUROC (100.00±0.00%) that was recorded most likely indicates overfitting, and therefore the results should be interpreted with great caution. The MUI-CNN variants show very wide CIs on Linux (MUI-CNN: macro F1 of 58.74±34.74%; MUI-CNN-z: macro F1 of 70.25±30.91%), indicating extreme fold-to-fold variability. The Informer encoder introduces instability, with folds 1, 2 and 4 systematically returning a zero AUROC for this sensor where per-row features are sufficient, and temporal context adds noise rather than signals. This might as well be the consequences of stratification, which disrupts row order and affects the positional encoding computed per sequence length of data. MUI-CNN-m achieves 72.80±17.93% with a narrower interval on macro F1, suggesting that MAD-based masking partially suppresses the noisy attention patterns on this sensor type.

**On the Network sensor**, all models achieve relatively tight CIs, confirming stable performance. MUI-CNN-m achieves the best macro F1 (90.67±1.94%), followed closely by MUI-CNN-z (macro F1 of 90.44±2.85%) and BiGRU-MHA (macro F1 of 88.73±5.15%). The overlapping CIs between MUI-CNN variants and BiGRU-MHA confirm that the performance gap on Network is not statistically decisive—bidirectional temporal modeling captures most of the discriminative structure present in network flow sequences.

**Windows OS sensors**—DCNN and T-DCNN achieve the tightest and highest macro F1 scores on both Windows datasets: Win7 macro F1 of 83.93±0.81% and 84.05±0.93%; and 81.65±1.53% and 80.69±1.41% for Win10. The extremely narrow CIs confirm that row-level OS telemetry features discriminate attack types consistently across all folds. Recurrent models show substantially wider intervals of macro F1 for the Win7 dataset (LSTM: 45.86±14.92%; BiLSTM: 51.97±14.26%), revealing high fold sensitivity that stems from the temporal clustering of minority attack classes within the Windows telemetry recording periods.

#### Per-Class Detection Analysis

Table 6 provides prediction performance, per class, per model, mean F1 scores across all sensors per dataset. It also displays the model performance for the Network dataset as a standalone for ease of comparability with the CIC IIoT dataset, due to their shared characteristics. It follows the same structure as Table 5. The main objective is to see how each model performs at each class detection and discuss the results accordingly. Table 7 instead summarizes per-class detection performance across all model combinations for both datasets, reporting mean F1 scores including zero-detection for sensor combinations. Including zeros is essential: a zero F1 is not a missing value but an informative detection failure, and excluding it would overstate the reliability of attack detection across the benchmark. The attack class column contains all categories of attacks per dataset; Support is the number of data points or windows used per model; Overall F1 contains the mean F1 score ± the confidence interval (CI) across all models and sensor combinations; MUI-CNN F1 represents the mean F1 score ± CI for all the MUI-CNN models with all masking variants; and Baseline F1 contains mean F1 score ± CI for all the other models used for comparison: DCNN, Temporal DCNN, LSTM, BiLSTM, BiGRU-MHA and the base Informer model.

**DoS** reaches an overall F1 of 73.5±10.1% (MUI-CNN: 85.6±5.6%; baselines: 65.4±13.0%), with the widest baseline CI reflecting inconsistent discrimination across IoT sensors of the TON IoT dataset. MUI-CNN* (i.e., MUI-CNN, MUI-CNN-z, and MUI-CNN-m, but excluding RDMD) narrows this further to 88.0±4.4%, the tightest CI in the All Sensors group, confirming that the CNN front-end stabilizes volumetric signature detection across sensors. On TON-IoT Network all architectures converge: DoS reaches 98.8±0.1% overall, with essentially no inter-model variance, which could be indicative of training issued. On the CIC IIoT 2025 dataset, DoS and DDoS are similarly strong: DoS reaches an overall F1 of 86.3±11.6%, with MUI-CNN* at 94.5±0.8% and the baseline at 90.7±4.2%, while DDoS reaches 85.1±13.1% overall, with MUI-CNN* at 93.7±1.2% and the baseline at 90.6±4.5%). The large gap between MUI-CNN* and MUI-CNN (79.6±29.3% for DoS; 76.8±33.1% for DDoS) isolates the RDMD collapse on this dataset, which failed to converge, reducing the MUI-CNN* group mean down by 15–17% and increasing the CI by a factor of 30–35. The baseline F1 is substantially higher relative to TON-IoT All Sensors. Under stratified cross-validation, windows are assigned to folds as atomic units, preserving intra-window temporal coherence. The sinusoidal positional encoding therefore supplies genuine local temporal context to the CNN front-end. The smaller MUI-CNN advantage on CIC relative to TON-IoT All Sensors reflects the pre-computed flow-level statistical structure of CIC rows: volumetric and rate features that the CNN would otherwise construct across rows within a window are already explicit single-row inputs, reducing the marginal value of convolutional feature extraction.

**DDoS** on TON-IoT All Sensors shows the sharpest architecture split in the table: baseline models average 58.1±13.5%, while MUI-CNN variants reach 83.9±17.3% (best single model: MUI-CNN-z at 94.4%), a gap of 25.8 percentage points. The split follows the presence of a temporal convolutional component: T-DCNN (the only baseline with a temporal CNN front-end) reaches 91.5%, while DCNN without temporal extension scores 45.3% and all attention and recurrent baselines (LSTM 49.0%, BiLSTM 51.0%, BiGRU 53.9%, and Informer 57.8%) cluster in the 45–58% range. This confirms that temporal convolutional feature extraction, not recurrence or attention alone, is necessary for DDoS discrimination across the heterogeneous sensor suite.

**Backdoor, injection, and password** achieve the highest MUI-CNN advantage on TON-IoT All Sensors. Backdoor overall F1 is 61.5±19.0% (MUI-CNN: 81.6±30.3%; baselines: 48.1±19.4%); injection 58.4±18.3% (MUI-CNN: 79.0±26.5%; baselines: 44.6±18.9%); password 60.6±17.8% (MUI-CNN: 80.9±25.5%, baselines: 47.1±18.3%). In all three cases the best individual MUI-CNN model reaches 92.9% (injection, MUI-CNN-z), 94.8% (password, MUI-CNN-m), and 97.1% (backdoor, MUI-CNN-m), with T-DCNN the only baseline achieving comparable scores (91.8%, 92.9%, and 97.3%, respectively—the sole baseline outperforming MUI-CNN variants on backdoor).

**Ransomware** is the only class where a baseline model outperforms all MUI-CNN variants on TON-IoT All Sensors: T-DCNN reaches 87.1%, versus MUI-CNN-z 85.6%, MUI-CNN-r 85.0%, MUI-CNN-m 85.4%, with the CNN Informer base model at 35.4%, reducing the MUI-CNN group mean down to 72.9±24.5%. Notably, MUI-CNN* (i.e., excluding MUI-CNN-r) at 68.8±32.7% is lower still, because RDMD (85.0%) contributes positively on this class. On TON-IoT, the Network class is the most challenging at 59.8±3.7% overall (MUI-CNN*: 63.7±4.1%, baselines: 57.5±5.2%), with all models detecting it consistently but struggling to distinguish it from bulk-transfer traffic at the flow level.

**XSS and scanning** are the classes most sensitive to the front-end architecture. On TON-IoT All Sensors, XSS reaches only 44.4±17.1% overall: pure recurrent models score 18.6% (LSTM, BiLSTM) to 25.3% (BiGRU), Informer 24.8%, while MUI-CNN variants reach 77.7–78.2%, a gap exceeding 50 points. Scanning at 40.8±11.3% overall shows a similar split: LSTM—14.8%; BiLSTM—18.8%; BiGRU—21.9%; Informer—43.6%, versus MUI-CNN-z—63.3%; MUI-CNN-r—56.5%; MUI-CNN-m—56.3%. Both classes require detecting local temporal probe or payload patterns within a window. The temporal convolutional front-end (present in T-DCNN and all MUI-CNN variants) provides this extraction, as confirmed by DCNN without temporal extension scoring comparably to recurrent baselines (26.6% XSS, 29.9% scanning).

**MITM** is the hardest class across all datasets. On TON-IoT All Sensors the overall F1 is 17.1±4.3%, with even the best group (MUI-CNN*) reaching only 20.2±13.5%, the widest relative CI in the table. The best single model is MUI-CNN-m at 32.7%, while baseline recurrent models score 9.5–22.2% and Informer 12.6%. On TON-IoT Network MITM improves to 45.1±10.3% overall, detected by all models but with low precision and recall (range 20.2–66.7%, best: BiGRU at 66.7%). The contrast with CIC IIoT 2025, where MITM reaches 79.3±7.8% (MUI-CNN*: 83.2±0.6%; baselines: 81.8±9.0%), confirms that the TON-IoT MITM challenge is a data-distribution artefact. TON-IoT MITM is sparse: 1480 test instances in the All Sensors partition and 600 in the Network partition (5-fold aggregate), yielding fewer than 300 instances per fold on average, insufficient for stable per-fold F1 estimation. The consistent MUI-CNN* advantage in CI width across both datasets, particularly on DoS (MUI-CNN* ±4.4% against baseline ±13.0% on All Sensors; ±0.8% against ±4.2% on CIC) and MITM (MUI-CNN* ±0.6% against the baseline MUI-CNN, or ±9.0% on CIC), reinforces that the attention-masking mechanism produces more stable predictions across the cross-validation folds.

**MITM** is the hardest class across all datasets. On TON-IoT All Sensors the overall F1 is 17.1±4.3%, with even the best group (MUI-CNN*) reaching only 20.2±13.5%, the widest relative CI in the table. The best single model is MUI-CNN-m at 32.7%, while baseline recurrent models score 9.5–22.2% and Informer 12.6%. On TON-IoT Network MITM improves to 45.1±10.3% overall, detected by all models but with low precision and recall (range 20.2–66.7%, best: BiGRU at 66.7%). The contrast with CIC IIoT 2025, where MITM reaches 79.3±7.8% (MUI-CNN*: 83.2±0.6%, MUI-CNN baseline: 81.8±9.0%), confirms that the TON-IoT MITM challenge is a data-distribution artifact: TON-IoT MITM is sparse, with 1480 test instances in the All Sensors partition and 600 in the Network partition (5-fold aggregate), yielding fewer than 300 instances per fold on average, which is insufficient for stable per-fold F1 estimation, while the CIC IIoT dataset provides 1471 instances distributed across folds, enabling reliable classification. The consistent MUI-CNN* advantage in CI width across both datasets, particularly on DoS (MUI-CNN* ±4.4% against the baseline ±13.0% on All Sensors; ±0.8% against ±4.2% on CIC) and MITM (MUI-CNN* ±0.6% against baseline ±9.0% on CIC), shows the benefit of the attention-masking mechanism.

**CIC IIoT 2025.** Overall performance is comparable to the TON-IoT Network for high-support classes, confirming that flow-level features generalize well across both benchmarks under the same training pipeline. The Reconnaissance dataset yields F1 of 88.5±17.9% overall (MUI-CNN*: 99.2±0.0%, baselines: 96.9±1.5%), web 88.5±15.1% (MUI-CNN*: 96.2±1.8%, baselines: 96.1±3.3%), and malware 93.5±6.7% (MUI-CNN*: 98.5±0.8%, baselines: 95.8±3.9%), all at 100% detection rate, with tight MUI-CNN* CIs consistent with TON-IoT Network. The sole exception is Brute Force at 61.7±20.7% overall, where baselines outperform MUI-CNN* (69.9±30.0% against 62.3±4.4%); however, only 364 test instances per-model scores range from 0% (for BiGRU) to 100.0% (LSTM), indicating fold-level instability rather than a systematic architectural signal, as confirmed by the wide baseline CI (±30.0%). The RDMD collapse dominates the MUI-CNN (including the MUI-CNN-r case) column for CIC. There is a decline of 8–23 percentage points below MUI-CNN*, while the CIs grow to ±15–45%, which is why the MUI-CNN* column is the benchmark for this dataset.

Performance conclusions for minority attack classes must be interpreted with caution. MITM is the most extreme case: instances are concentrated in very few sensors and the per-fold validation support is insufficient for stable F1 estimation, such that a small number of misclassifications produces large variations in the results. Scanning on individual IoT sensors faces a similar constraint, with several model–sensor combinations scoring zero F1 due to insufficient fold support rather than actual detection failure. All per-class F1 values are computed from pooled out-of-fold predictions across all five folds to maximize effective test support; results for these sparse classes should be treated as indicative rather than conclusive. This is a structural limitation of the class distribution in the TON-IoT benchmark, not a pipeline artifact.

Considered together, the per-class results reveal a consistent stratification: classes with high fold support and volumetric or payload signatures detectable within a single window (DoS, DDoS, scanning on Network) reach near-ceiling F1 across all architectures, while classes that are either rare (MITM, Ransomware) or require cross-sensor generalization (XSS, scanning on IoT sensors) expose the performance gap between the temporal convolutional front-end and pure recurrent or attention baselines. The MUI-CNN* group narrows this gap on every high-support class and consistently produces tighter CIs, indicating that the attention-masking mechanism improves not only the mean performance but also the prediction stability across cross-validation folds, a property that is as operationally relevant as the point estimate for deployment in heterogeneous IoT environments.

### 5.3. Component Ablation via Mixed-Effects Regression

To isolate the independent contribution of each architectural component relative to the flat MLP (DCNN and T-DCNN) and recurrent (LSTM, BiLSTM, and BiGRU-MHA) baseline models, a mixed-effects linear regression was fitted with per-class F1 score as the response variable. Binary indicators for each component (CNN front-end, Informer encoder, MAD masking, RDMD masking, and Z-score masking) were included as fixed influences, taking the value of zero for all baseline models and one when the corresponding component is present. Fold was included as a random intercept to account for the repeated-measures structure of 5-fold cross-validation. The inverse support coefficient $n_{i}$ for the *i*-th class was used as observation weight ($w_{i}=1/n_{i}$), following the rationale that F1 score estimates for rare attack classes carry higher variance than those for the more frequent attack classes, to ensure fairness in the detection of all attack types regardless of their prevalence. This weighting approach is similar to the use of macro-averaged F1 as the main evaluation metric and ensures that the regression coefficients reflect model performance on rare as well as common attack classes equally. The intercept, therefore, estimates the mean per-class F1 of the baseline group, and each coefficient quantifies the independent additive contribution of the corresponding component above that baseline. Table 8 reports the estimated contribution of each component with CIs of 95% as well as *p*-values.

**CNN Front-end:** This is the dominant architectural component, contributing an estimated +30.3 percentage points for the F1 ($p<10^{-36}$) independently of all other components. Informer encoder scoring +10.7 points, for $p<10^{-8}$. Together, these two core components account for +41 points for the F1 over the baseline, directly confirming the necessity of both local convolutional feature extraction and global temporal attention for multi-class attack detection across heterogeneous IoT sensor streams.

**Encoder Masking:** Among the masking strategies, MAD and Z-score masking each contribute marginally significant +4.7–4.8 F1 points in aggregate (p<0.05), while RDMD masking does not reach significance (p=0.526, CI crosses zero). The non-significance of RDMD in aggregate does not preclude sensor-specific utility: as shown in Table 5, RDMD achieves the highest macro F1 on GPS (91.21±3.79%) and Weather (93.18±4.12%), but its gains on those sensors are offset by near-zero effects elsewhere. This pattern confirms that masking strategy selection is best guided by the statistical distribution of the target sensor stream rather than a single global choice.

The CIC IIoT 2025 and TON-IoT Network partitions differ substantially in scale, with 30,030 flow-level windows versus 2,234,386 (75 times more) raw sensor rows respectively, yet macro-averaged F1 scores across model groups remain comparable. Model overall average performance on TON-IoT Network data is 87.7±3.0% (Baseline: 87.2±3.4%, MUI-CNN: 88.4±5.4%, MUI-CNN*: 87.6±7.2%), while CIC IIoT 2025 reaches 84.9±12.4% overall (Baseline: 90.1±7.2%, MUI-CNN: 77.1±27.2%, MUI-CNN*: 91.0±1.2%). The increased MUI-CNN confidence interval (CI) on CIC IIoT (±27.2%) is attributable entirely to the RDMD convergence failure on that dataset; once excluded, MUI-CNN* produces the tightest CI between both datasets (±1.2%), narrower even than the TON-IoT Network baseline (±3.4%). This stability at 75 times smaller sample size and with double the number of features confirms that the pipeline’s performance hierarchy is mostly governed by architecture and feature representation rather than driven by the training set size, and that the compact, pre-aggregated flow format characteristic of lightweight edge capture agents is sufficient for reliable detection. It also indicates that, depending on the detection target, a smaller sample of the network data, for instance 1% sample of the data (approximately 1/100 packet), could be enough to achieve reasonable detection target while maintaining the computational cost within the capabilities of edge IoT devices.

### 5.4. Architectural Limitations

A direct comparison between encoder-only and full encoder–decoder Informer configurations is not included in the present study, as the encoder-only design was adopted a priori following the established practice for classification tasks [9]. This represents a limitation of the current ablation and is identified as a direction for future work. Similarly, the contribution of the universal embedding layer in isolation—without the CNN or Informer components—is partially evidenced by the DCNN results that use the same embedding but without sequence modeling, though a dedicated embedding-only ablation would provide stronger evidence.

Overall, the statistical masking layer’s contribution is most clearly demonstrated on sensors for which the minority attack classes produce low-amplitude temporal variations. For the Thermostat data type, the DCNN without masking scores an F1 of 0% for the scanning data type across 24 test instances. The MUI-CNN-m achieves the highest F1 of 95.81% for the Thermostat data samples, while for the GarageDoor data, the zscore-masked MUI-CNN achieves a macro F1 of 96.96% versus 87.64% for the MUI-CNN-r variant. These results validate the masking mechanism’s design rationale—they suppress dominant statistical patterns and direct encoder attention toward minor local variations that contain early-warning information. The selection of the appropriate masking method can be done either based on expert empirical knowledge of the data on which detection is applied, or by applying a set of masking methods and proceeding with a voting mechanism as outlined in our research paper [9], which describes an ensemble voting approach. This is recommended by default.

### 5.5. Computational Efficiency

Efficiency is assessed across four dimensions relevant to IoT edge deployment: theoretical complexity (MMACs, parameter count, and model size), inference latency and throughput, CPU and Random Access Memory (RAM) utilization during testing, and training cost. All timing and memory figures are averaged over about 2–5 epochs of a single fold to exclude first-epoch CUDA warm-up overhead, and are reported as minimum–maximum ranges across the 11 TON-IoT sensors and overall on CIC-IoT-2025 (Table 9).

#### 5.5.1. Architectural Complexity

The DCNN and T-DCNN are by far the lightest architectures, requiring only 0.04–0.07 MMACs and 42–70 thousand parameters per sample, with a model file of 0.16–0.27 MB. Their complexity grows only marginally with *F* because the MLP operates on a flat feature vector. LSTM requires 13–16 MMACs and 199–255 thousand parameters (0.76–0.97 MB), while BiGRU-MHA reaches 14.7 MMACs and 214–228 parameters (0.82–0.87 MB). BiLSTM roughly doubles the LSTM complexity to 34–41 MMACs and 528–642 thousand parameters (2.01–2.45 MB) due to the model’s bidirectional processing.

The Informer and MUI-CNN family models are the most computationally intensive group at 440 MMACs and 591–605 thousand parameters (2.25–2.31 MB). Masking strategies (MAD, Z-score, and RDMD) add zero parameters and zero MMACs to any MUI-CNN variant, as masking is applied to the input before the encoder, and is not reapplied during the testing, the identical MMACs and model sizes across all MUI-CNN variants confirm this.

#### 5.5.2. Inference Latency and Throughput

DCNN and T-DCNN achieve a mean inference time of 0.0034 ms and 0.0033 ms per sample respectively, with a throughput of 298,000–299,000 samples/s, by far the fastest of all architectures. This is a direct consequence of their flat-vector MLP design—without a recurrent loop or attention mechanism, the performance scales linearly with batch size and processes packets in under 4 μs each.

The recurrent models form a middle tier. BiGRU-MHA achieves 0.013 ms and ≈82,000 samples/s, LSTM reaches 0.014 ms and ≈73,000 samples/s, and BiLSTM performs at 0.023 ms and ≈44,000 samples/s due to its wider bidirectional hidden state. All three remain well above the 10,000 samples/s threshold needed for real-time classification of typical IoT traffic.

The transformer-based architectures are the slowest at testing but still achieve latency below 0.07 ms per sample. The standalone Informer averages 0.026 ms (≈40,000 samples/s) and the unmasked MUI-CNN 0.028 ms (≈36,000 samples/s), 7.6 and 8.5 times slower, respectively, than the DCNN. Among the MUI-CNN masking variants, Z-score and MAD add only 5–8% overhead over the unmasked baseline MUI-CNN (0.030 ms and 0.031 ms), whereas RDMD imposes a substantial penalty (0.057 ms, ≈18,000 samples/s), making it the slowest model. On the Network sensor specifically, the Informer sustains 29,000 samples/s and MUI-CNN 27,000 samples/s, versus 303,000 samples/s for the DCNN, illustrating that the DCNN’s performance is most pronounced on high-volume traffic.

#### 5.5.3. CPU Utilization and RAM at Inference

The CPU utilization during testing is low across all architectures. On the Network sensor, every model uses only 3–12% of total machine CPU capacity, leaving the majority of processor resources available for concurrent system processes. This confirms that inference latency is governed by memory bandwidth and sequence length rather than raw compute, and that none of the architectures would contend for CPU time on a shared edge host.

Peak RAM during testing scales with dataset size rather than model architecture. Small structured IoT sensors (Fridge, Modbus, Thermostat, and MotionLight) require 1.47–1.75 GB of memory regardless of the chosen model, driven primarily by the Python 3.12.4 and PyTorch 2.5 runtime overhead. The Linux sensor requires ≈ 3.3 GB and the Network sensor ≈ 5.1 GB. Model architecture affects RAM by at most 10–15% within the same sensor, confirming that RAM is determined by the data volume loaded into memory rather than the model parameters. At 0.16 MB (DCNN) to 2.31 MB (MUI-CNN family), all model weights are negligible relative to the runtime memory floor of ≈1.5 GB, so the RAM does not make a difference between architectures for deployment planning.

For deployment on single-board-computer-class edge devices (Raspberry Pi 4, NVIDIA Jetson Nano: 4–8 GB RAM), all models are viable for inference on small-to-medium IoT sensors. The Network and Linux sensors require 3.3 = −5.1 GB, and are therefore suitable only for the higher-memory tier (i.e., ≥8 GB). Micro-controller class devices (ESP32, Arduino: <8 MB) are incompatible with any PyTorch-based model without quantization and framework replacement.

#### 5.5.4. Training Cost

Training cost is very architecture dependent, showing that DCNN and T-DCNN are by far taking the longest time to train, requiring a mean of 5.86 s per epoch across TON-IoT sensors (range 0.26–20.73 s, driven by sensor size). All recurrent and transformer models are trained much faster: LSTM in 0.21 s on average (0.01–0.76 s) and BiGRU-MHA in 0.25 s, while the MUI-CNN variants take 0.88–1.09 s. For the Network sensor dataset, the DCNN requires 20.7 s per epoch versus 4.4 s for the unmasked MUI-CNN (reduction of 6.6 times), and 4.0 s for the standalone Informer (7.3 less time). Among the MUI-CNN variants, it is the RDMD that adds the most training overhead (1.09 s mean, 5.06 s for the Network sensor), while Z-score and MAD take the same time as the unmasked MUI-CNN (0.88 s).

The efficiency profiles on the CIC-IoT-2025 dataset (F=81 features) are consistent with TON-IoT. T-DCNN achieves the fastest testing at 0.0036 ms and 281,000 samples/s, with the DCNN following at 0.0038 ms and 261,000 samples/s. Recurrent models range from 0.017 ms (BiGRU-MHA, 59,000 samples/s) to 0.027 ms (BiLSTM, 36,000 samples/s). The Informer reaches 0.030 ms and MUI-CNN variants 0.031–0.058 ms, with the RDMD variant again the slowest at 0.058 ms (17,000 samples/s). The relative ordering across all models is identical to TON-IoT, confirming that the efficiency hierarchy is architecture driven rather than dataset specific.

#### 5.5.5. IoT Device Suitability for Deployment

Table 10 summarizes testing deployment suitability for MUI-CNN across five representative IoT device classes, characterized by RAM capacity, processor generation, floating-point capability, and weight storage. Two constraints determine feasibility. First, *weight storage*—all evaluated models fit within 2.31 MB and are accommodated by the flash memory of even the most constrained micro-controller, making weight storage a non-binding constraint across every device class. Second, the *activation memory*—during inference, the network must hold the intermediate feature maps produced at each layer in the RAM before they are consumed by the next. For the Informer self-attention encoder within MUI-CNN, these intermediate matrices scale with sequence length and model dimension, requiring an estimated 23–116 MB per forward pass depending on the sensor feature dimension *F*; i.e., one to two orders of magnitude beyond the memory of 512 KB–8 MB available on micro-controller class hardware. Equally critical is the presence of a *Floating-Point Unit* (FPU)—without dedicated floating-point silicon, each multiply-accumulate operation is emulated in software at a delay of tens to hundreds of cycles, rendering transformer testing latency impractical. All 64-bit ARM Cortex-A processors (ARMv8-A), present from the low-end Single Board Computer (SBC) tier upward, include a full hardware FPU and Single Instruction, Multiple Data (SIMD) extensions that accelerate the parallel vector operations central to attention-based inference.

Considering both constraints together, the table shows that MUI-CNN testing is feasible from the mid-range SBC tier upward: devices with 2–8 GB of RAM and a 64-bit ARM processor running at 1.5–1.8 GHz, such as gateway-class or industrial IoT hardware, provide sufficient activation memory and floating-point throughput. Industrial IoT gateways, with RAM ranging from 1 to 8 GB and embedded Multi Media Card (eMMC) storage of 8–32 GB, represent the most operationally realistic deployment target for MUI-CNN in constrained field environments. DCNN and T-DCNN remain the only feasible architectures on micro-controller class nodes, albeit at the cost of near-random multi-class classification on structured IoT sensors, as reported in the overall performance section. It should also be noted that for network traffic datasets such as TON-IoT and CIC-IIoT-2025, data are collected centrally on the server infrastructure rather than on individual edge nodes. Training on such data is necessarily executed centrally and edge inference constraints do not apply to that sensor group.

Across all sensors and datasets, efficiency divides into three tiers. The DCNN and T-DCNN offer maximum testing speed (≈298,000 samples/s, <0.004 ms/sample, <0.27 MB model) at the cost of the slowest training (5.86 s/epoch mean, up to 20.7 s on the Network sensor). Recurrent models (BiGRU-MHA, LSTM, and BiLSTM) form a middle tier: 4–7 times slower at inference than the DCNN but 16–28 times faster to train, with compact model sizes of 0.76–2.45 MB. Transformer-based models (Informer and MUI-CNN variants) are the slowest at testing (0.026–0.057 ms/sample) yet 6–7 faster to train than the DCNN. Among the MUI-CNN variants, Z-score and MAD impose negligible testing overhead over the unmasked baseline (<10%), while the RDMD doubles the inference latency and should be used only when its anomaly-suppression properties justify the cost. RAM is dominated by the runtime environment rather than model weights across all architectures. For real-time edge deployment on single-board computers, all models are feasible for small-to-medium IoT sensors, with the DCNN and T-DCNN being preferable when latency is the primary constraint, while the Informer and MUI-CNN variants are preferable when training speed and detection accuracy on complex traffic profiles are of greater priority.

## 6. Conclusions

This paper presented MUI-CNN, a Multi-modal Universal Informer CNN Classifier for multi-class attack detection across heterogeneous IoT sensor and network environments. The architecture delivers on four stated contributions. The Multi-modal Universal embedding layer enables a single model to operate across heterogeneous sensor types without dataset-specific preprocessing. The CNN front-end captures local spatial anomaly patterns, contributing +30.3 F1 points ($p<10^{-36}$) above the recurrent baseline. The encoder-only Informer captures long-term temporal dependencies through ProbSparse self-attention while eliminating the decoder to reduce complexity, contributing a further improvement of 10.7% for F1 (with $p<10^{-8}$). The optional Statistical Masking layer suppresses dominant spike patterns prior to encoding, with MAD and Z-score masking yielding statistically significant aggregate gains (4.7 and 4.8 higher F1 percentage points respectively, p<0.05) across the eleven TON-IoT sensors.

Together, these components enable MUI-CNN variants to achieve macro F1 scores of 75.4–97.2% on the five simple structured IoT sensors where all flat and recurrent baselines collapse below 19.5%, and above 90% on the Network sensor, while remaining competitive on high-dimensional OS sensors. The optimal masking strategy varies by sensor type, confirming that it should be treated as a sensor-specific hyperparameter. Ensemble or voting-based masking selection [9] are identified as directions for future work.

From a computational efficiency standpoint, the evaluated architectures divide into three tiers. DCNN and T-DCNN offer maximum inference speed (<0.004 ms/sample) but are the slowest to train and collapse on structured sensors. Recurrent models occupy a middle tier, 4–7 times slower at testing but 16–28 times faster to train. Among MUI-CNN masking variants, Z-score and MAD impose negligible inference overhead (<10%). Regarding deployment, MUI-CNN testing is adequate for mid-range SBC and IoT gateway hardware (≥2 GB RAM, 64-bit ARM with hardware FPU), as detailed in Table 10. For network traffic datasets, data are collected centrally and training is necessarily executed server side, so edge constraints do not apply to that sensor group. On-device retraining in response to concept drift is not feasible on constrained nodes. Centralized training with federated aggregation is identified as a direction for future work.

Remaining limitations include reduced injection detection on the Linux dataset (macro F1 58.3–79.5% for MUI-CNN versus 84.0% for DCNN), suggesting that per-row feature combinations are individually discriminative for OS telemetry and do not benefit from sequence modeling, or that further hyperparameter tuning is required. Scanning detection on low-feature-complexity sensors similarly remains unresolved. More broadly, the relationship between feature selection, input dimensionality, and computational cost has not been explored in this work: a sensitivity analysis examining how feature subsets and sampling strategies affect the accuracy–efficiency trade-off would clarify whether MUI-CNN’s computational complexity can be reduced without material loss in detection performance, and is identified as a direction for future work.

## Figures and Tables

**Table 1:** Summary of related works and contributions of the proposed approach.

Article	Method	Pros	Limitations	Corresponding Contribution of This Paper
[6,11] (2020)	ML-based outlier detection; Isolation Forest and KNN.	Grid-based anomaly detection with short-term candidate maintenance.	Delayed outlier classification. Short-term memory only. Numerical data only.	Long-term temporal dependency and pattern. Immediate detection. Supports multiple data types through the universal embedding layer.
[5] (2019)	Probability-based OD using a Copula model on multivariate numerical data.	Computationally efficient; reduces multivariate to univariate.	Numerical data only. No temporal correlation.	Supports multiple data types; accounts for temporal correlation.
[4] (2022)	VAE trained for normality learning and anomaly detection.	Unsupervised learning of normality.	Unable to differentiate attack types.	Learns and differentiates attack patterns. Higher detection accuracy.
[7] (2022)	Anomaly scoring via encoder-decoder and Informer in Intelligent Transport.	Improves anomaly detection in transport data.	Limited to univariate analysis.	Multivariate model; relies solely on the encoder layer.
[12] (2025), [13,14] (2024)	1D-CNN-LSTM for IoT network anomaly detection.	Spatial and long-term temporal anomaly detection.	Weaker for very long temporal forecasting.	Informer-based model performs better at very long-term forecasting.
[15] (2024)	LSTM, BiLSTM, GRU, and GRU with self-attention for IoT IDS on TON-IoT.	GRU with attention achieves 99% accuracy with 84% reduction in classification time.	Single recurrent architecture evaluated per model; no multi-sensor benchmark.	Systematic comparison of recurrent, convolutional, and transformer models across eleven heterogeneous IoT sensor streams.
[16] (2024), [17] (2025)	BiGRU with attention mechanism for IIoT and IoT intrusion detection on Edge-IIoTset and CIC IoT 2023.	Bidirectional temporal modeling; attention selectively weights critical timesteps; addresses class imbalance.	Single dataset evaluation; no multi-type sensor heterogeneity considered.	Evaluated on eleven heterogeneous sensor modalities including OS telemetry, network flows, and physical IoT sensors under stratified cross-validation.
[18] (2025), [19] (2025)	Hybrid BiLSTM-CNN and CNN-BiLSTM-Attention IDS for IoT network traffic.	Combines spatial CNN feature extraction with bidirectional temporal modeling.	Network traffic only; limited to binary or coarse-grained classification.	Multi-class attack-type classification across physical, OS, and network IoT sensors with universal embedding for heterogeneous feature types.
[20] (2025)	Hybrid GRU and BiLSTM for intrusion detection on TON-IoT and CIC-IDS-2018.	Complementary gating and bidirectional memory; Pearson-based feature selection.	Single model combination; no comparison with transformer-based architectures.	Benchmarks BiGRU Multi-Head Attention (MHA), BiLSTM, and LSTM alongside Informer and CNN-Informer variants, providing a unified architectural comparison on the same TON-IoT benchmark.
[10] (2023)	Temporal Convolution + Informer with RDMD and delayed perception.	Reduced false-positives; accuracy-focused.	Delayed detection; computationally complex.	Instant detection; universal embedding; reduced complexity.
[21] (2025), [22] (2022)	Base Informer for Intrusion Detection System (IDS) and DDoS.	Temporal features with fixed windows; efficient.	Global context only.	Conv1D adds local pattern recognition within data segments.
[23] (2026)	Informer + Entropy-Guided Gradient Pruning (EGGP).	Reduces noise; focuses on anomalous patterns.	Applicable to network data only.	Reduced complexity by removing the Informer decoder and adding masking.
[24] (2024)	GAT + Informer for short/long-term forecasting.	Short-term and long-term temporal correlation.	Increased complexity; no spatial patterns.	Identifies local spatial patterns and long-term temporal correlation.
[8] (2024)	Hybrid CNN-Informer for cloud network attack detection.	High detection performance; spatio-temporal patterns.	Higher complexity; unsupervised mode cannot classify attack types.	Reduced complexity with encoder-only Informer and masking. Supervised classification.
[9] (2025)	Informer encoder statistical masking for OD in univariate data.	Reduces complexity; improves learning.	Univariate numerical continuous data only.	Universal embedding supports multiple data types; improves performance by dropping the decoder.

**Table 2:** Dataset shape and attack rate by component and sensor.

Dataset	Component	Sensor	Records	Features (*F*)	Attack Rate (%)
**TON-IoT**	Telemetry	Fridge	587,076	7	14.7
		Garage Door	591,446	8	12.8
		GPS Tracker	595,686	18	13.3
		Modbus	287,194	10	22.4
		Motion Light	452,262	8	14.1
		Thermostat	442,228	8	12.7
		Weather	650,242	13	13.9
	OS	Linux	1,000,000	32	15.74
		Linux (Win env.)	1,000,000	32	14.55
		Windows 7	28,367	137	21.08
		Windows 10	35,975	136	31.86
	Network	Network	2,234,386	44	96.25
**CIC IIoT 2025**	Network & Logs	All devices	30,030	75	45.55

**Table 3:** Preprocessing treatment per sensor.

Dataset	Sensor	Null/Missing	Temporal Encoding	Num.	Cat.	Bool.	Presence Flags
**TON-IoT**	Weather	N/A	Sin/cos 6 cycles	7	—	—	—
	Fridge	N/A	Sin/cos 6 cycles	1	—	1	—
	GarageDoor	N/A	Sin/cos 6 cycles	—	2	—	—
	Thermostat	N/A	Sin/cos 6 cycles	1	—	1	—
	Modbus	N/A	Sin/cos 6 cycles	4	—	—	—
	MotionLight	N/A	Sin/cos 6 cycles	—	1	1	—
	GPS	lat=0, lon=10 flagged	Sin/cos 6 cycles	12	—	1	—
	Network	Replace “–” and NaN with 0	Sin/cos 6 cycles	17	5	7	9
	Linux	N/A	Sin/cos 6 cycles	23	3	—	—
	Win7	Replace “–”/“space” with 0	Sin/cos 6 cycles	131	—	—	—
	Win10	Replace “–”/“space” with 0	Sin/cos 6 cycles	130	—	—	—
**CIC IIoT**	Network & Logs (aggregated)	Replace ±∞ with 0	Sin/cos 6 cycles	68	1	—	—

**Table 4:** Hyperparameter configuration per model. RLR-P—Reduce Learning Rate on Plateau (RLR-P); Robust—RobustScaler (quantile range of 5.95). All CNN-Informer masking variants share the same hyperparameters.

Hyperparameter	DCNN	T-DCNN	BiGRU-MHA	BiLSTM	LSTM	Informer	MUI-CNN
Architecture	MLP	MLP	BiGRU + MHA	BiLSTM	LSTM	Transformer	CNN-Transf.
Input type	numerical, categorical, boolean features	numerical, categorical, boolean, temporal features	Sequences	Sequences	Sequences	Sequences	Sequences
Sequence length	—	—	64	64	64	64	64
Hidden layers	256 → 128 → 64	256 → 128 → 64	128	128	128	128	128
Attention heads	—	—	4	—	—	8	8
Transformer layers	—	—	—	—	—	3	3
LSTM/GRU layers	—	—	2	2	2	—	—
Dropout	0.3/0.2	0.3/0.2	0.2	0.3	0.3	0.1	0.1
Batch size	64	64	32	32	32	32	32
Learning rate	$1\times 10^{-3}$	$1\times 10^{-3}$	$1\times 10^{-4}$	$1\times 10^{-4}$	$1\times 10^{-4}$	$1\times 10^{-3}$	$1\times 10^{-3}$
Optimizer	Adam	Adam	Adam	Adam	Adam	Adam	Adam
Weight decay	—	—	—	—	—	$1\times 10^{-5}$	$1\times 10^{-5}$
Learning rate scheduler	RLR-P	RLR-P	—	—	—	RLR-P	RLR-P
Scheduler patience	2	2	—	—	—	2	2
Scheduler factor	0.5	0.5	—	—	—	0.5	0.5
Epoch ceiling	50	50	50	50	50	50	50
ES patience (*P*)	5	5	5	5	5	5	5
ES threshold (δ)	$10^{-4}$	$10^{-4}$	$10^{-4}$	$10^{-4}$	$10^{-4}$	$10^{-4}$	$10^{-4}$
Gradient clip	1.0	1.0	1.0	1.0	1.0	1.0	1.0
Scaler	Robust	Robust	Robust	Robust	Robust	Robust	Robust
Scaler IQR range	5–95%	5–95%	5–95%	5–95%	5–95%	5–95%	5–95%
Feature clip	±10	±10	±10	±10	±10	±10	±10
CV folds	5	5	5	5	5	5	5

**Table 5:** Cross-validation accuracy (%), AUROC (%), and macro-averaged F1 (%) by model and sensor. Best result per sensor and metric in **bold**. Temporal DCNN is abbreviated to T-DCNN, and MAD/Z-score/RDMD masking are denoted as MUI-CNN-m/z/r.

	Sensor	Metric	DCNN	T-DCNN	LSTM	BiLSTM	BiGRU-MHA	Informer	MUI-CNN	MUI-CNN-z	MUI-CNN-r	MUI-CNN-m
**TON-IoT**	Weather	Accuracy	80.68	81.48	85.51	84.79	93.16	99.00	98.94	**99.06**	**99.06**	98.96
		AUROC	81.68	81.50	72.12	82.43	96.56	**99.84**	99.78	99.82	99.83	99.69
		macro F1	37.48	37.48	26.17	37.84	70.46	89.32	89.23	92.76	**93.00**	90.82
	Fridge	Accuracy	85.31	85.31	85.23	85.23	85.23	85.32	99.19	99.09	**99.20**	99.06
		AUROC	49.94	49.95	49.79	49.69	50.13	49.98	**99.89**	99.87	99.88	99.86
		macro F1	13.15	13.15	13.15	13.15	13.15	13.15	**97.22**	96.61	97.05	97.06
	Thermostat	Accuracy	87.27	87.27	87.26	87.26	87.16	87.29	99.50	99.48	99.45	**99.54**
		AUROC	50.78	50.90	49.66	50.34	59.50	52.18	99.87	**99.91**	99.84	99.86
		macro F1	13.31	13.31	13.31	13.31	13.76	13.32	**89.88**	89.50	82.01	83.90
	MotionLight	Accuracy	85.86	85.86	85.91	85.91	85.81	85.89	98.38	**98.58**	98.47	98.49
		AUROC	50.04	49.98	50.58	50.39	55.99	50.16	**99.84**	99.83	99.82	99.78
		macro F1	11.55	11.55	11.55	11.55	11.76	11.55	87.63	**89.45**	87.85	87.48
	GarageDoor	Accuracy	87.87	87.87	82.80	82.52	80.80	85.19	**99.13**	99.09	98.99	**99.13**
		AUROC	89.65	89.64	91.63	91.19	92.70	93.57	**99.87**	99.84	99.85	**99.87**
		macro F1	16.13	16.13	16.54	16.49	18.85	19.49	92.22	92.29	91.33	**93.21**
	Modbus	Accuracy	77.60	77.60	77.52	77.52	77.52	77.58	96.21	**96.35**	95.78	95.95
		AUROC	50.44	50.36	50.43	50.07	54.42	50.00	98.70	99.02	**99.10**	98.84
		macro F1	14.56	14.56	14.56	14.56	14.56	14.56	75.37	81.49	79.88	**81.67**
	GPS	Accuracy	87.92	87.93	93.88	94.69	95.14	81.72	98.90	**99.12**	98.98	98.86
		AUROC	96.42	96.41	97.86	98.22	98.62	84.70	99.84	**99.86**	99.84	99.80
		macro F1	69.18	69.41	58.75	61.20	66.57	20.36	89.51	90.69	**90.92**	89.65
	Network	Accuracy	96.59	95.50	98.22	98.46	98.65	98.90	**99.23**	99.20	99.20	99.20
		AUROC	99.79	99.76	99.88	99.94	99.91	**99.97**	**99.97**	**99.97**	**99.97**	**99.97**
		macro F1	84.00	81.60	84.24	86.47	89.61	88.57	90.22	90.59	**90.81**	90.65
	Linux	Accuracy	99.68	**99.69**	87.01	89.48	88.76	86.70	94.05	96.89	93.50	98.17
		AUROC	**100.00**	**100.00**	96.69	98.33	98.03	96.72	99.38	99.80	97.16	99.82
		macro F1	**83.96**	82.52	28.44	39.35	36.04	33.81	58.90	69.55	58.27	79.47
	Win10	Accuracy	**97.55**	97.18	92.79	93.69	92.43	95.52	95.07	95.61	95.43	95.61
		AUROC	**99.70**	99.67	98.31	98.95	98.62	98.93	98.95	98.94	98.96	99.11
		macro F1	**81.61**	80.66	59.23	63.75	63.34	72.80	73.16	73.64	73.96	73.70
	Win7	Accuracy	98.93	**98.95**	92.18	94.48	91.95	96.25	96.59	96.59	96.25	96.25
		AUROC	**99.79**	99.78	97.76	97.92	97.56	98.80	99.07	99.33	99.05	98.72
		macro F1	83.93	**84.04**	48.23	53.95	51.74	69.56	71.31	71.67	70.05	70.80
**CIC IIoT**	All devices	Accuracy	96.61	96.66	95.87	94.78	92.61	96.13	**97.10**	**97.20**	55.16	96.88
		AUROC	99.70	99.70	99.29	99.43	99.45	99.71	**99.88**	99.77	61.62	99.85
		macro F1	94.81	**94.85**	94.43	91.80	76.11	88.62	90.69	91.75	35.50	90.39

**Table 6:** Per-class F1 (%) per model per dataset. Best result per sensor in bold. Temporal DCNN is abbreviated to T-DCNN and MAD/Z-score/RDMD denoted as MUI-CNN-m/z/r.

			Baseline						MUI-CNN			
Dataset	Class	Support (Aggregates)	DCNN	T-DCNN	LSTM	BiLSTM	BiGRU	Informer	MUI-CNN	MUI-CNN-z	MUI-CNN-r	MUI-CNN-m
TON-IoT—All Sensors	Normal	9,016,964	93.6	98.0	92.2	92.9	93.0	93.9	93.9	98.1	97.9	**98.1**
	DDoS	1,571,832	45.3	91.5	49.0	51.0	53.9	57.8	57.6	**94.4**	89.6	93.9
	DoS	827,820	61.5	76.6	49.3	54.4	58.2	**92.6**	92.4	85.0	78.4	86.5
	Scanning	1,583,191	29.9	59.1	14.8	18.8	21.9	43.6	43.6	**63.3**	56.5	56.3
	MITM	1480	15.1	18.0	9.5	13.3	22.2	12.6	9.0	18.8	19.8	**32.7**
	Injection	259,842	37.9	91.8	26.9	33.4	37.8	39.8	38.4	**92.9**	92.2	92.5
	Password	760,075	40.0	92.9	29.7	36.1	41.5	42.3	41.8	93.8	93.0	**94.8**
	Ransomware	51,523	25.7	**87.1**	19.8	22.7	25.1	35.5	35.4	85.6	85.0	85.4
	XSS	480,709	26.6	72.1	18.6	18.6	25.3	24.8	24.7	77.7	76.9	**78.2**
	Backdoor	657,971	40.0	**97.3**	35.4	38.5	41.9	35.6	35.2	97.1	97.1	97.0
TON-IoT–Network	Normal	79,633	89.1	90.0	77.5	83.6	83.5	93.0	**93.0**	89.3	89.5	89.5
	DDoS	616,500	99.8	99.8	99.5	99.5	99.6	97.5	97.2	99.8	**99.8**	99.8
	DoS	337,532	98.8	98.9	98.6	98.8	98.8	**99.0**	98.6	99.0	98.9	99.0
	Scanning	714,015	99.9	**99.9**	99.7	99.7	99.8	97.8	97.6	99.9	99.9	99.9
	Injection	45,625	94.7	**99.7**	93.7	93.1	95.1	82.5	69.8	99.6	99.5	99.7
	MITM	600	45.2	54.1	28.6	40.0	**66.7**	22.3	20.2	56.4	59.5	58.1
	Password	171,856	99.5	**99.8**	98.0	98.3	98.8	93.3	87.1	99.8	99.8	99.8
	Ransomware	7280	60.3	60.0	48.5	53.6	55.3	67.2	**67.9**	62.2	61.4	61.1
	XSS	210,894	98.9	99.9	98.9	99.2	99.2	95.9	95.5	**100.0**	99.9	99.9
	Backdoor	50,811	99.6	**99.9**	99.3	98.9	99.3	91.5	89.1	99.9	99.9	99.8
CIC IIoT 2025	Normal	13,680	98.0	97.9	**100.0**	99.8	98.8	99.8	100.0	99.9	68.3	100.0
	DDoS	3234	97.4	**97.5**	86.8	84.3	88.2	89.4	94.5	94.0	26.2	92.5
	DoS	3280	96.4	**96.6**	87.8	84.3	86.5	92.7	95.1	94.7	34.6	93.8
	Recon	6005	95.0	95.0	97.1	97.1	97.1	**100.0**	99.2	99.2	6.4	99.2
	Malware	1444	95.4	95.7	**100.0**	100.0	86.8	96.8	97.7	98.9	64.7	98.9
	MITM	1471	92.8	**93.0**	83.7	80.0	62.5	78.7	82.6	83.5	52.6	83.5
	Web	552	95.4	95.1	**100.0**	100.0	88.9	97.1	97.1	97.1	20.0	94.4
	Brute Force	364	88.1	87.9	**100.0**	88.9	0.0	54.5	59.3	66.7	11.1	60.9

**Table 7:** Per-class mean F1 ± 95% confidence interval across model groups per dataset. Best result per sensor is presented in bold. MUI-CNN* F1 combines the results of MUI-CNN, MUI-CNN-m, and MUI-CNN-z but not MUI-CNN-z as the RDMD masking failed to converge with CIC-IIoT.

Dataset	Class	Support (Aggregates)	Overall F1	Baseline F1	MUI-CNN F1	MUI-CNN* F1
TON-IoT–All Sensors	Normal	9,016,964	95.1 ±1.6	93.9 ±1.7	**97.0** ±2.0	96.7 ±2.7
	DDoS	1,571,832	68.4 ±13.0	58.1 ±13.5	**83.9** ±17.3	82.0 ±23.9
	DoS	827,820	73.5 ±10.1	65.4 ±13.0	85.6 ±5.6	**88.0** ±4.4
	Scanning	158,3191	40.8 ±11.3	31.4 ±13.6	**54.9** ±8.1	54.4 ±11.3
	MITM	1480	17.1 ±4.3	15.1 ±3.6	20.1 ±9.5	**20.2** ±13.5
	Injection	259,842	58.4 ±18.3	44.6 ±18.9	**79.0** ±26.5	74.6 ±35.5
	Password	760,075	60.6 ±17.8	47.1 ±18.3	**80.9** ±25.5	76.8 ±34.3
	Ransomware	51,523	50.7 ±19.0	36.0 ±20.5	**72.9** ±24.5	68.8 ±32.7
	XSS	480,709	44.4 ±17.1	31.0 ±16.3	**64.4** ±25.9	60.2 ±34.8
	Backdoor	657,971	61.5 ±19.0	48.1 ±19.4	**81.6** ±30.3	76.4 ±40.4
TON-IoT–Network	Normal	79,633	87.8 ±3.0	86.1 ±4.5	90.3 ±1.8	**90.6** ±2.4
	DDoS	616,500	99.2 ±0.6	**99.3** ±0.7	99.2 ±1.3	99.0 ±1.7
	DoS	337,532	98.8 ±0.1	98.8 ±0.1	**98.9** ±0.2	98.8 ±0.3
	Scanning	714,015	99.4 ±0.6	**99.5** ±0.6	99.3 ±1.1	99.2 ±1.5
	Injection	45,625	92.7 ±6.0	**93.1** ±4.6	92.2 ±14.6	89.7 ±19.5
	MITM	600	45.1 ±10.3	42.8 ±13.1	**48.5** ±18.6	44.9 ±24.2
	Password	171,856	97.4 ±2.6	**98.0** ±1.9	96.6 ±6.2	95.6 ±8.3
	Ransomware	7280	59.8 ±3.7	57.5 ±5.2	63.1 ±3.1	**63.7** ±4.1
	XSS	210,894	98.7 ±1.0	98.7 ±1.1	**98.8** ±2.2	98.5 ±2.9
	Backdoor	50,811	97.7 ±2.5	**98.1** ±2.6	97.2 ±5.3	96.2 ±7.0
CIC IIoT 2025	Normal	13,680	96.2 ±6.1	99.0 ±0.8	92.0 ±15.5	**100.0** ±0.1
	DDoS	3234	85.1 ±13.1	90.6 ±4.5	76.8 ±33.1	**93.7** ±1.2
	DoS	3280	86.3 ±11.6	90.7 ±4.2	79.6 ±29.3	**94.5** ±0.8
	Recon	6005	88.5 ±17.9	96.9 ±1.5	76.0 ±45.5	**99.2** ±0.0
	Malware	1444	93.5 ±6.7	95.8 ±3.9	90.1 ±16.6	**98.5** ±0.8
	MITM	1471	79.3 ±7.8	81.8 ±9.0	75.6 ±15.0	**83.2** ±0.6
	Web	552	88.5 ±15.1	96.1 ±3.3	77.2 ±37.4	**96.2** ±1.8
	Brute Force	364	61.7 ±20.7	**69.9** ±30.0	49.5 ±25.3	62.3 ±4.4

**Table 8:** Mixed-effects ablation: estimated contribution of each architectural component to per-class macro F1. Random intercept on fold.

Component	Estimate	SE	CI*_low_*	CI*_high_*	*p*-Value
CNN front-end	+0.303	0.024	0.257	0.350	$1.27\times 10^{-36}$ *
Informer encoder	+0.107	0.018	0.071	0.143	$6.10\times 10^{-9}$ *
MAD masking	+0.047	0.024	0.000	0.093	0.049 **
Z-score masking	+0.048	0.024	0.002	0.095	0.041 ***
RDMD masking	+0.015	0.024	−0.031	0.062	0.526

*p*-value: * p<0.001; ** p<0.01; *** p<0.05.

**Table 9:** Computational efficiency by model and dataset. Best result per metric per dataset in **bold**. TON-IoT values are minimum–maximum ranges across 11 sensors and overall CIC-IoT-2025. MMACs and model size vary with the input feature dimension *F*, throughput and testing time per sample, and training time per epoch.

	Metric	DCNN	T-DCNN	LSTM	BiGRU	BiLSTM	Informer	MUI-CNN	MUI-CNN-z	MUI-CNN-r	MUI-CNN-m
**TON-IoT**	MMACs	0.04–0.07	0.04–0.07	12.65–16.29	14.68–14.70	33.69–40.96	440.42–441.33	440.42–441.33	440.42–441.33	440.42–441.33	440.42–441.33
	Size (MB)	0.16–0.27	0.16–0.27	0.76–0.97	0.82–0.87	2.01–2.45	2.25–2.31	2.25–2.31	2.25–2.31	2.25–2.31	2.25–2.31
	Tput (K/s)	279–306	**281–308**	50–81	53–91	33–48	29–45	27–41	26–38	15–19	26–37
	Train/ep (s)	0.26–20.73	0.27–20.97	**0.01–0.76**	0.01–0.89	0.02–1.32	0.03–3.99	0.04–4.37	0.04–4.30	0.05–5.06	0.04–4.32
	Inf (ms/smp)	0.0032–0.0036	**0.0032–0.0036**	0.0124–0.0201	0.0110–0.0189	0.0208–0.0305	0.0224–0.0342	0.0247–0.0365	0.0266–0.0388	0.0520–0.0666	0.0272–0.0391
**CIC-IoT**	MMACs	0.06	0.06	14.94	14.69	38.27	440.99	440.99	440.99	440.99	440.99
	Size (MB)	0.23	0.23	0.89	0.85	2.29	2.29	2.29	2.29	2.29	2.29
	Tput (K/s)	261	**281**	55	59	36	34	32	31	17	29
	Train/ep (s)	0.28	0.28	**0.01**	0.01	0.02	0.03	0.03	0.04	0.05	0.04
	Inf (ms/smp)	0.0038	**0.0036**	0.0182	0.0168	0.0274	0.0295	0.0311	0.0328	0.0579	0.0340

**Table 10:** Inference deployment suitability by IoT device class. All evaluated models produce weights ≤2.31 MB, the binding constraint for transformer-class architectures is activation memory during the forward pass, estimated at 23–116 MB for MUI-CNN variants, depending on sensor feature dimension *F*.

Device Class	RAM	Processor	Weight Storage	MUI-CNN Inference Feasible?
Micro-controller	512 KB–8 MB	32-bit ARM; 200–480 MHz; limited or no FPU	≤16 MB flash	**No**—limited activation RAM; no FPU for attention layers
Low-end SBC	512 MB	64-bit ARM; ∼1 GHz; hardware FPU	microSD	**Marginal**—Sufficient RAM, limited throughput
Mid-range SBC	2–8 GB	64-bit ARM; 1.5–1.8 GHz; hardware FPU	microSD/eMMC	**Yes**
IoT Gateway/AI SBC	4 GB	64-bit ARM; 1.4–1.5 GHz; integrated GPU	eMMC 16 GB	**Yes**—hardware acceleration available
Industrial IoT Gateway	1–8 GB	64-bit ARM; 1.5–1.8 GHz; hardware FPU	eMMC 8–32 GB	**Yes**

## Data Availability

The raw data supporting the conclusions of this article will be made available by the authors on request.

## Abbreviations

The following abbreviations are used in this manuscript:

AUROC	Area Under the Receiver Operating Characteristic
BiGRU	Bidirectional Gated Recurrent Unit
BiLSTM	Bidirectional Long Short-Term Memory
CI	Confidence Interval
CIS	Canadian Institute for Cybersecurity
CNN	Convolutional Neural Network
Conv1D	1D Convolutional Neural Network
CPU	Central Processing Unit
CSV	Comma-Separated Values
CV	Cross-Validation
DCNN	Deep Convolutional Neural Network
DDoS	Distributed Denial of Service
DoS	Denial of Service
EGGP	Entropy-Guided Gradient Pruning
ES	Early Stopping
FC	Fully Connected
FFN	Feed-Forward Network
GAT	Graph Attention Transformer
GPU	Graphical Processing Unit
GPS	Global Positioning System
GRU	Gated Recurrent Unit
IDS	Intrusion Detection System
IIoT	Industrial Internet of Things
IoT	Internet of Things
IQR	Interquartile Range
KNN	K-Nearest Neighbors
LSTM	Long Short-Term Memory
MACs	Multiply-Accumulate Operations
MAD	Median Absolute Deviation
MCD	Minimum Covariance Determinant
MHA	Multi-Head Attention
MITM	Man-in-the-Middle
ML	Machine Learning
MMACs	Million Multiply-Accumulate Operations
MLP	Multi-Layer Perceptron
MUI-CNN	Multi-modal Universal Informer Convolutional Neural Network
NaN	Not a Number
OS	Operating System
PCAP	Packet Capture
ProbSparse	Probabilistic Sparse
RDMD	Robust Dynamic Mahalanobis Distance
RF	Random Forest
RLR-P	Reduce Learning Rate on Plateau
SBC	Single-Board Computer
SIMD	Single Instruction, Multiple Data
SVM	Support Vector Machine
TON	Telemetry, Operating Systems, and Network
VAE	Variational Auto-Encoder
WSN	Wireless Sensor Network
XSS	Cross-Site Scripting

## References

[1] Mishra R., Mishra A. (2025). Current research on Internet of Things (IoT) security protocols: A survey. Comput. Secur..

[2] Zhou H., Zhang S., Peng J., Zhang S., Li J., Xiong H., Zhang W. (2021). Informer: Beyond efficient transformer for long sequence time-series forecasting. Proc. AAAI Conf. Artif. Intell..

[3] Zhou H., Li J., Zhang S., Zhang S., Yan M., Xiong H. (2023). Expanding the prediction capacity in long sequence time-series forecasting. Artif. Intell..

[4] Gouda W., Tahir S., Alanazi S., Almufareh M., Alwakid G. (2022). Unsupervised outlier detection in IoT using deep VAE. Sensors.

[5] Ghalem S.K., Kechar B., Bounceur A., Euler R. (2019). A probabilistic multivariate copula-based technique for faulty node diagnosis in wireless sensor networks. J. Netw. Comput. Appl..

[6] Bhatti M.A., Riaz R., Rizvi S.S., Shokat S., Riaz F., Kwon S.J. (2020). Outlier detection in indoor localization and Internet of Things (IoT) using machine learning. J. Commun. Netw..

[7] Peng X., Lin Y., Cao Q., Cen Y., Zhuang H., Lin Z. Traffic Anomaly Detection in Intelligent Transport Applications with Time Series Data Using INFORMER. Proceedings of the 2022 IEEE 25th International Conference on Intelligent Transportation Systems (ITSC).

[8] Yuan H., Wang S., Bi J., Zhang J., Zhou M. (2024). Hybrid and spatiotemporal detection of cyberattack network traffic in cloud data centers. IEEE Internet Things J..

[9] Mfondoum R.N., Gotseva N., Vlahov A., Ivanov A., Koleva P., Poulkov V., Manolova A. (2025). Mask inflation encoder and quasi-dynamic thresholding outlier detection in cellular networks. Telecom.

[10] Chen W., Zhou H., Cheng L., Xia M. (2023). Condition monitoring and anomaly detection of wind turbines using temporal convolutional Informer and robust dynamic Mahalanobis distance. IEEE Trans. Instrum. Meas..

[11] Zhu R., Ji X., Yu D., Tan Z., Zhao L., Li J., Xia X. (2020). KNN-based approximate outlier detection algorithm over IoT streaming data. IEEE Access.

[12] Sasi T., Lashkari A.H., Lu R., Xiong P., Iqbal S. (2025). An efficient self attention-based 1D-CNN-LSTM network for IoT attack detection and identification using network traffic. J. Inf. Intell..

[13] Dharshiniya S., S D.M.R. (2024). IoT network intrusion detection with deep learning and voice alerts. Proceedings of the 2024 International Conference on IoT Based Control Networks and Intelligent Systems (ICICNIS), Bengaluru, India, 17–18 December 2024.

[14] Ding W., Abdel-Basset M., Mohamed R. (2023). DeepAK-IoT: An effective deep learning model for cyberattack detection in IoT networks. Inf. Sci..

[15] Mutleg M.L., Mahmood A.M., Jawad Al-Nayar M.M. (2024). Deep Learning Based Intrusion Detection System of IoT Technology: Accuracy Versus Computational Complexity. Int. J. Saf. Secur. Eng..

[16] Yang K., Wang J., Li M. (2024). An improved intrusion detection method for IIoT using attention mechanisms, BiGRU, and Inception-CNN. Sci. Rep..

[17] Bilal M., Zeng R., Muhammad O., Adil M., Shoukat S. (2025). A cyber-resilient and explainable intrusion detection system for Internet of Things networks. Clust. Comput..

[18] Sadhwani S., Modi U.K., Muthalagu R., Pawar P.M. (2026). A hybrid BiLSTM-CNN approach for intrusion detection for IoT applications. Sci. Rep..

[19] Nandanwar H., Katarya R. (2026). IA-IDS: An intelligent adaptive intrusion detection system for IoT security using CNN, BiLSTM, and attention mechanism. Peer -Peer Netw. Appl..

[20] Nadhum K.A., Sam S.M., Yusuf S. (2025). A Deep Learning Algorithm to Cybersecurity: Enhancing Intrusion Detection with a Hybrid GRU and BiLSTM Model. Eng. Technol. Appl. Sci. Res..

[21] Cui W., Liao X., Yang Y., Feng S., Song M. (2025). Informer-based DDoS attack detection method for the power Internet of Things. PLoS ONE.

[22] Sun Y., Hou L., Lv Z., Peng D. (2022). Informer-based intrusion detection method for network attack of integrated energy system. IEEE J. Radio Freq. Identif..

[23] Raju Y., Nagappa P.C. (2026). Entropy-guided gradient pruning with Informer for intrusion detection system in Internet of Things. J. Artif. Intell. Technol..

[24] Zhao M., Peng H., Li L., Ren Y. (2024). Graph attention network and Informer for multivariate time series anomaly detection. Sensors.

[25] Mahalanobis P.C. (1936). On the generalised distance in statistics. Proc. Nat. Inst. Sci. India.

[26] Rousseeuw P.J., Driessen K.V. (1999). A fast algorithm for the minimum covariance determinant estimator. Technometrics.

[27] Moustafa N. (2021). A new distributed architecture for evaluating AI-based security systems at the edge: Network TON_IoT datasets. Sustain. Cities Soc..

[28] Canadian Institute for Cybersecurity (2025). CIC IIoT Dataset 2025. https://www.unb.ca/cic/datasets/iiot-dataset-2025.html.

[29] Bouckaert R.R., Frank E. (2004). Evaluating the replicability of significance tests for comparing learning algorithms. Proceedings of the Pacific-Asia Conference on Knowledge Discovery and Data Mining.

